# Using Phylogenetic and Coalescent Methods to Understand the Species Diversity in the *Cladia aggregata* Complex (Ascomycota, Lecanorales)

**DOI:** 10.1371/journal.pone.0052245

**Published:** 2012-12-18

**Authors:** Sittiporn Parnmen, Achariya Rangsiruji, Pachara Mongkolsuk, Kansri Boonpragob, Aparna Nutakki, H. Thorsten Lumbsch

**Affiliations:** 1 Department of Biology, Srinakharinwirot University, Bangkok, Thailand; 2 Department of Biology, Ramkhamhaeng University, Bangkok, Thailand; 3 Botany Department, The Field Museum, Chicago, Illinois, United States of America; 4 Department of Biological Sciences, University of Chicago, Chicago, Illinois, United States of America; New York State Health Department and University at Albany, United States of America

## Abstract

The *Cladia aggregata* complex is one of the phenotypically most variable groups in lichenized fungi, making species determination difficult and resulting in different classifications accepting between one to eight species. Multi-locus DNA sequence data provide an avenue to test species delimitation scenarios using genealogical and coalescent methods, employing gene and species trees. Here we tested species delimitation in the complex using molecular data of four loci (nuITS and IGS rDNA, protein-coding GAPDH and Mcm-7), including 474 newly generated sequences. Using a combination of ML and Bayesian gene tree topologies, species tree inferences, coalescent-based species delimitation, and examination of phenotypic variation we assessed the circumscription of lineages. We propose that results from our analyses support a 12 species delimitation scenario, suggesting that there is a high level of species diversity in the complex. Morphological and chemical characters often do not characterize lineages but show some degree of plasticity within at least some of the clades. However, clades can often be characterized by a combination of several phenotypical characters. In contrast to the amount of homoplasy in the morphological characters, the data set exhibits some geographical patterns with putative species having distribution patterns, such as austral, Australasian or being endemic to Australia, New Zealand or Tasmania.

## Introduction

Ever since Darwin's seminal study on the origin of species was published, the issue of delimiting species as the fundamental taxonomic unit has fascinated evolutionary biologists [1]–[3]. Understanding the delimitation of species is crucial for numerous fields of biological and ecological sciences and is important for conservation issues. However, the main challenge is finding and using appropriate character sets and analytical methods to recognize species. This is especially true for organisms with relatively simple morphologies, such as fungi where it is often difficult to find sufficient defining characters, or the choice of which might be viewed as arbitrary. Traditionally, species circumscriptions in lichen-forming fungi are based on phenotypical characters, such as thallus and ascomatal morphology and anatomy, or chemical characters, such as presence of extrolites (secondary metabolites). Environmental factors have been shown to influence phenotypic characters in various groups of lichens [4]–[6]. Also a remarkable amount of morphological disparity within clades has been demonstrated in several clades of lichenized fungi [7]–[13] calling the use of these characters to delimit taxa in question. Furthermore, it has repeatedly been shown that the traditional species delimitation underestimates the diversity of these fungi with numerous cryptic lineages discovered under currently accepted species in various unrelated families [14]–[39].

Here we focus on a notoriously difficult group of lichenized fungi, the *Cladia aggregata* complex. Previous classifications have accepted between one [40] and eight species [5] based on different interpretations of morphological and chemical diversity in the group. The genus is especially diverse in Tasmania with a number of chemo- and morphotypes only known from this island [5]. The group occurs in all vegetation types in Tasmania, including coastal heathland, wet peatlands, sclerophyll forests, rainforests, and alpine vegetation. Within Tasmania, much of the chemical diversity is confined to the south-west of the island. This area is rich in Tasmanian endemics and austral species with restricted geographical distributions [41]. Hence, from a conservational point of view, it is important to understand whether the bewildering morphological and chemical variation is due to a single, highly polymorphic species, or whether these represent distinct clades with restricted distributions.

The genus *Cladia* belongs to Cladoniaceae (Lecanorales, Ascomycota), which currently includes 16 genera with over 400 accepted species [42]. Most genera in this family have a dimorphic thallus with a crustose or foliose primary thallus and a vertical secondary thallus that bears fruiting bodies [43]–[45]. A few genera in the family also have foliose thalli [46]–[50]. *Cladia* spp. have a crustose primary thallus and a fruticose, secondary thallus, often referred to as pseudopodetium. In addition to the species with dimorphic thallus, our previous studies demonstrated that foliose taxa earlier classified in *Heterodea* and the crustose *Ramalinora glaucolivida* also belong here [7], [8], [51]. Currently, 14 species are accepted in *Cladia* [5], [51], [52]. In addition to a remarkable morphological variation, there is an unusually high chemical diversity as well. Seven major chemosyndromes have been detected in the group [5], [52]. Some of these chemotypes have been accepted as species [5], [53], while other authors regard them as chemical races of a polymorphic species [40], [54], [55]. In previous phylogenetic studies [7], [8], we found *Cladia aggregata* to be non-monophyletic, although the taxon sampling in these studies was insufficient to address the species delimitation in the group. These findings and the fact that there is no consensus on the delimitation of species in the group prompted this study.

To address the species delimitation in the *C. aggregata* complex, we have generated a data set using four loci: internal transcribed spacer of nuclear ribosomal DNA (ITS), intergenic spacer of nuclear ribosomal DNA (IGS), and the protein-coding genes nuclear glyceraldehyde-3-phosphate dehydrogenase (GAPDH) and DNA replication licensing factor Mcm7 (Mcm7). The molecular data were used to perform gene trees in a maximum likelihood (ML) and Bayesian (B/MCMC) framework and coalescent-based species trees. We employed a combination of methods to address species delimitation in the *Cladia aggregata* complex, using gene tree estimation from single-locus and concatenated data sets, and species tree estimations. As a conservative first estimate we used a genealogical species recognition approach [56] in which presence of clades in the majority of single-locus genealogies is taken as evidence that these represent distinct lineages [57]–[59]. Since recently derived species will remain undiscovered by this method due to incomplete lineage sorting [60], [61], we additionally used coalescent-based approaches. The general mixed Yule coalescent (GMYC) method developed by Pons *et al*. [62] and Monaghan *et al*. [63] allows, without prior expectations, locating of nodes that define the transitions between intraspecific (tokogenetic) and interspecific relationships. This method uses branch lengths differences to identify nodes that circumscribe species. A number of studies have employed this coalescent approach to successfully delineate species in various groups of organisms [63]–[71]. To choose among different species delimitation scenarios supported by the genealogical and GMYC methods, we employed an approach that utilizes a species tree estimation method to inform species delimitation decisions by a likelihood ratio test that measures the fit of gene trees within a given species tree. Finally, we re-examined morphological and chemical characters of the sequenced samples to study their phylogenetic structure in order to identify characters to distinguish these lineages.

Specifically, we addressed the following research questions: 1) does the *Cladia aggregata* complex represent a single, phenotypically variable species or multiple species-level lineages?, 2) is the phenotypical variation randomly distributed within the complex or correlated to phylogenetic structure, and 3) is there a correlation of geographical distribution and phylogenetic structure?.

## Results

### Phylogenetic analyses

Four hundred and eighty-six DNA sequences of 126 representative samples of four loci were assembled, including 474 newly generated sequences (table S1). The specimens included the seven chemosyndromes in the *Cladia aggregata* complex and four of the currently described species in addition to *C. aggregata*, viz. *C. deformis, C. dumicola, C. inflata*, and *C. moniliformis*. We have not been able to get fresh material of *C. mutabilis* and *C. oreophila* and have not been able to obtain sequences from herbarium material of these species from HO. The latter species is a rare taxon that is only known from a few localities in remote mountain ranges of south-western Tasmania, whereas *C. mutabilis* is restricted to high rainfall peatlands in south-western Tasmania. We included only samples for which sequences for at least three of the four loci were generated in the analysis.

Table 1 includes summary statistics for the four single-locus data sets. The single locus ML trees are shown in the figure S1. Since no strongly supported conflicts between the four single-locus ML phylogenetic trees were detected, a combined data set was analyzed. In the B/MCMC analysis of the combined data set, the likelihood parameters in the sample had a mean likelihood of LnL  = −10,728.85 (±0.354), while the ML tree had a likelihood of LnL = −9,763.28.

**Table 1:** Sequence characteristics of sampled markers used in the present study.

	IGS	ITS	*GAPDH*	*Mcm7*
Aligned length (bp)	396	520	793	510
Newly generated sequences	122	118	122	112
Variable characters	57	32	71	36
Model selected	GTR+I+G	SYM+G	GTR+G	GTR+G

Including alignment length (number of base pairs); variable sites for each sampled locus; and locus-specific model of evolution identified using the Akaike information criterion in JMODELTEST.

**Figure 1:**
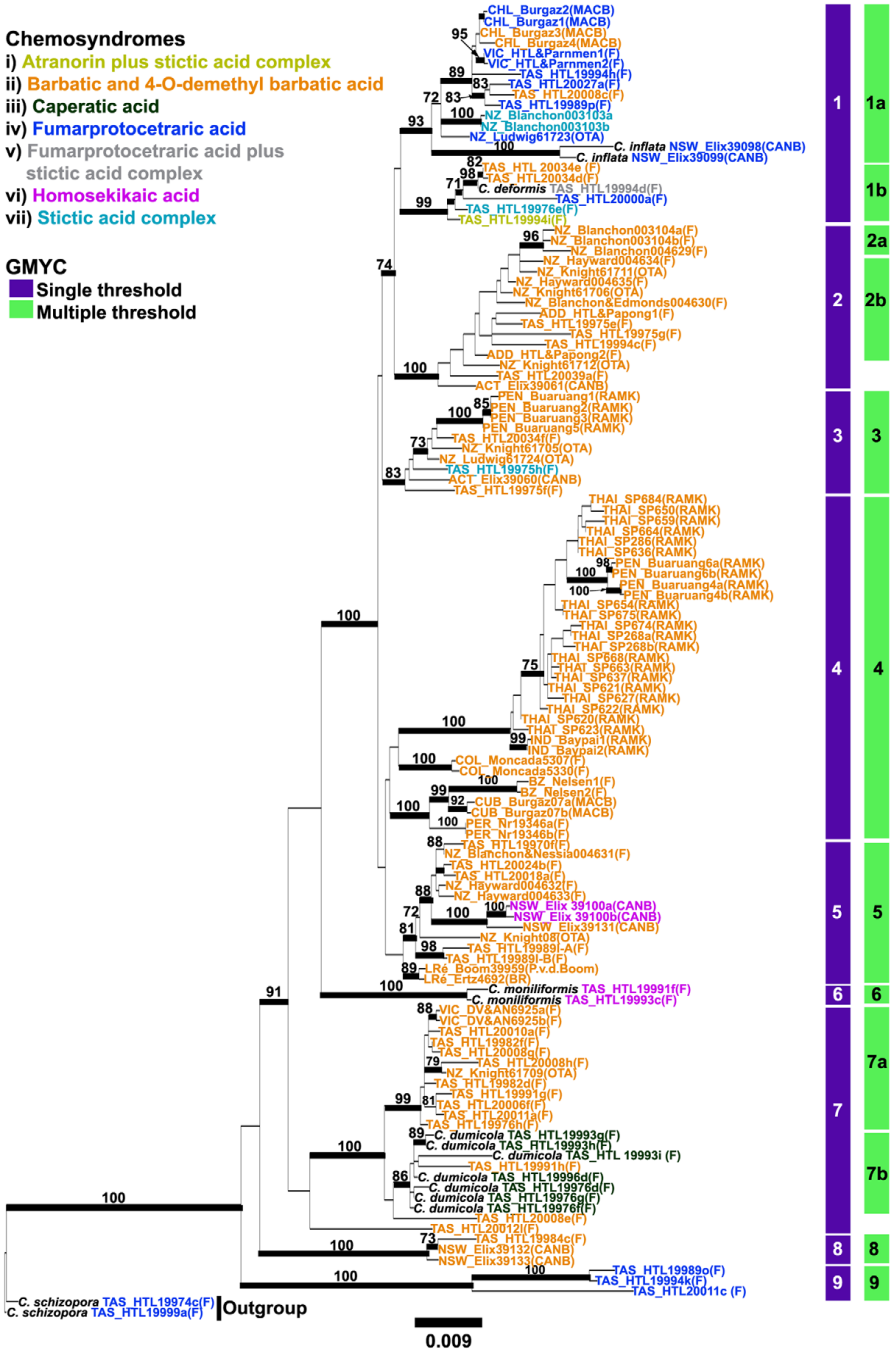
Maximum Likelihood tree depicting relationships within the *Cladia aggregata* complex on the basis of a concatenated data set including nuITS, IGS, protein-coding GAPDH and protein -coding Mcm7 sequences and estimated using partitioned ML analysis. Bootstrap support equal or above 70% is shown (ML) and posterior probabilities equal or above 0.95 are indicated as bold branches. The purple (single threshold) and green (multiple thresholds) shades represented species recognized by GMYC method.

The phylogenetic estimates of the ML and B/MCMC analyses were congruent; hence only the ML tree (Fig. 1) is shown here. The *Cladia aggregata* s. lat. samples fall into several clades (Fig. 1). The clades discussed below are those identified by the GMYC analyses as discussed below. Some clades consist of strongly supported subclades. There are some associations between clades and chemotypes as well as geographical origins. Six clades (3, 4, 5, 6, 8 & 9) are recognized by both methods of GMYC, whereas three clades (1, 2 & 7) are not identical in the single- and multiple-thresholds GMYC modes. Of the nine clades obtained from the single-threshold, three have restricted distributional ranges (clades 6, 8 & 9). Clades 6 and 9 are restricted to Tasmania. Clade 8 includes specimens from mainland Australia and Tasmania. Five clades correspond with specific chemotypes (clades 2, 4, 6, 8 & 9). Clades 2, 4 and 8 include only members containing the barbatic acid chemosyndrome. Clade 9 includes samples with the fumarprotocetraric acid chemosyndrome. Clade 6 includes specimens with the homosekikaic acid chemosyndrome. The multiple-threshold mode of GMYC recognized 12 clades. Five clades corresponded to their geographical origins (1b, 2a, 6, 8 & 9). Clades 1b, 6 and 9 were restricted to Tasmania. Clade 2a included specimens from New Zealand, whereas clade 8 was composed of specimens from mainland Australia and Tasmania. Seven clades contained specimens with a specific chemosyndrome (Clades 2a, 2b, 4, 6, 7a, 8 & 9). Clades 2a, 2b, 4, 7a and 8 contained only members with the barbatic acid chemosyndrome, while the homosekikaic and fumarprotocetraric acids chemosyndromes were found in clades 6 and 9, respectively.

Clade 1 includes samples from three currently accepted species, viz. *C. aggregata, C. inflata* and *C. deformis*. This clade has an austral distribution, occurring in Australia, New Zealand and southern South America (southern part of Chile). Two samples of *C. inflata* are clustering together and are nested within this clade, whereas *C. deformis* forms sister subclades and is clustering with *C. aggregata*. Five chemotypes are found in this clade, including the atranorin plus stictic, barbatic, fumarprotocetraric, fumarprotocetraric plus stictic and stictic acid chemosyndromes. However, the multiple-threshold mode recognized two distinct clades (1a & 1b) within this clade. Clade 2 consisted entirely of the members currently placed in *C. aggregata* containing the barbatic acid chemosyndrome and occurring in Australasia (including New Zealand). The multiple-threshold mode separated two different groups (2a & 2b). Clade 2a included only samples from New Zealand. Clade 3 included *C. aggregata* from Australasia and Southeast Asia (Penang). This clade comprised species containing the barbatic acid and stictic acid chemosyndromes. Clade 4 included the samples of the *C. aggregata* from Southeast Asia (Thailand and Malaysia), India and Neotropics. This clade comprised species containing the barbatic acid chemosyndrome. Clade 5 included *C. aggregata* samples from Australasia and La Réunion, which possess either the barbatic acid or homosekikaic acid chemosyndrome. Two morphologically distinct samples of *C. moniliformis* form clade 6 and contain the homosekikaic acid chemosyndrome. So far, *C. moniliformis* is only known from Tasmania. Clade 7 comprises specimens from Tasmania containing either the barbatic acid or caperatic acid chemosyndrome. While the majority of barbatic acid containing samples in this clade form one subclade, the other subclade included only two barbatic acid containing samples, which are intermixed with the caperatic acid containing samples (*C. dumicola*). Interestingly, the multiple-threshold mode placed all *C. dumicola* samples into one putative species (7a & 7b). Clade 7a contained only the *C. aggregata* samples with barbatic acid, while clade 7b included mainly specimens of *C. dumicola*. Clade 8 contained specimens collected in Australia. The specimens in this clade contained the barbatic acid chemosyndrome. Tasmanian collections containing the fumarprotocetraric acid chemosyndrome formed clade 9.

### Species Delimitation based on the General Mixed Yule Coalescent (GMYC)

The GMYC model estimates the species delimitation from DNA surveys by identifying independently evolving lineages as a transition from intraspecific (coalescent mode) to interspecific (speciation branching patterns) relationships on a phylogenetic tree. The most recent common ancestral node at the transition point is interpreted as distinguishing species. 58 haplotypes were found in the combined data set. Identical haplotypes and the two outgroup samples (*C. schizopora*) were removed for the analysis. A maximum likelihood tree obtained from a RAxML search using the combined data set was used for the analyses. The GMYC analyses revealed different results for the single- and multiple-threshold modes.

**Figure 2:**
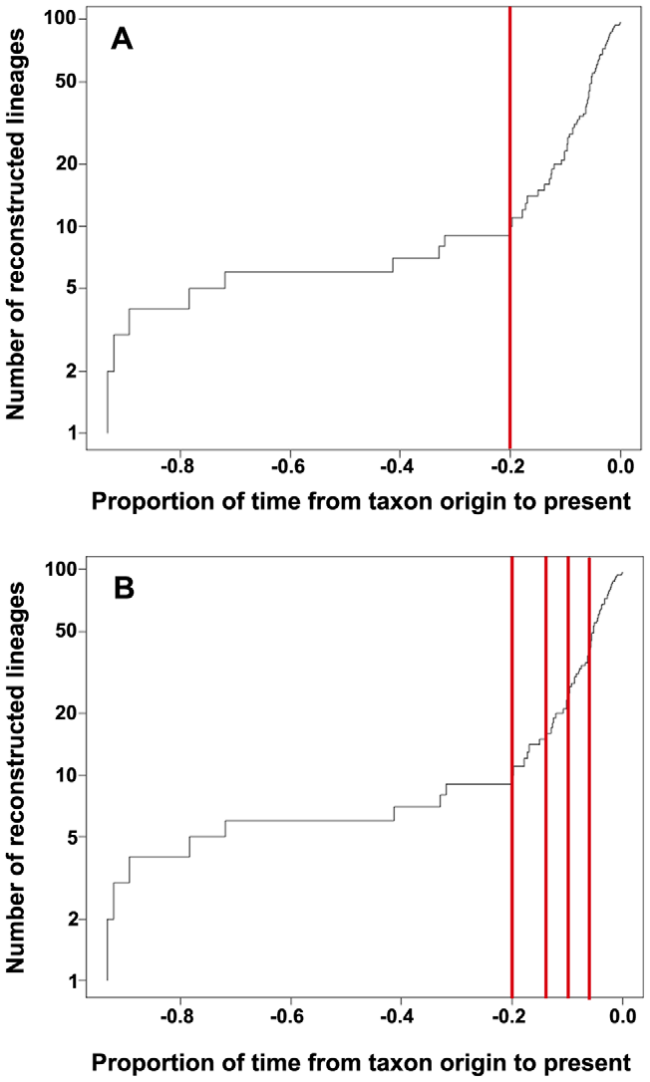
Lineage-through-time (LTT) plot for the *Cladia aggregata* group, including (A) single threshold analysis and (B) multiple threshold analysis of GMYC. Lines are actual numbers of reconstructed lineages for the clade. Time is expressed as a proportion of the total time since the first cladogenesis event inferred for the taxon. The sharp increase in branching rate, corresponding to the transition from interspecies to intraspecies branching events, is indicated by the red line(s).

**Figure 3:**
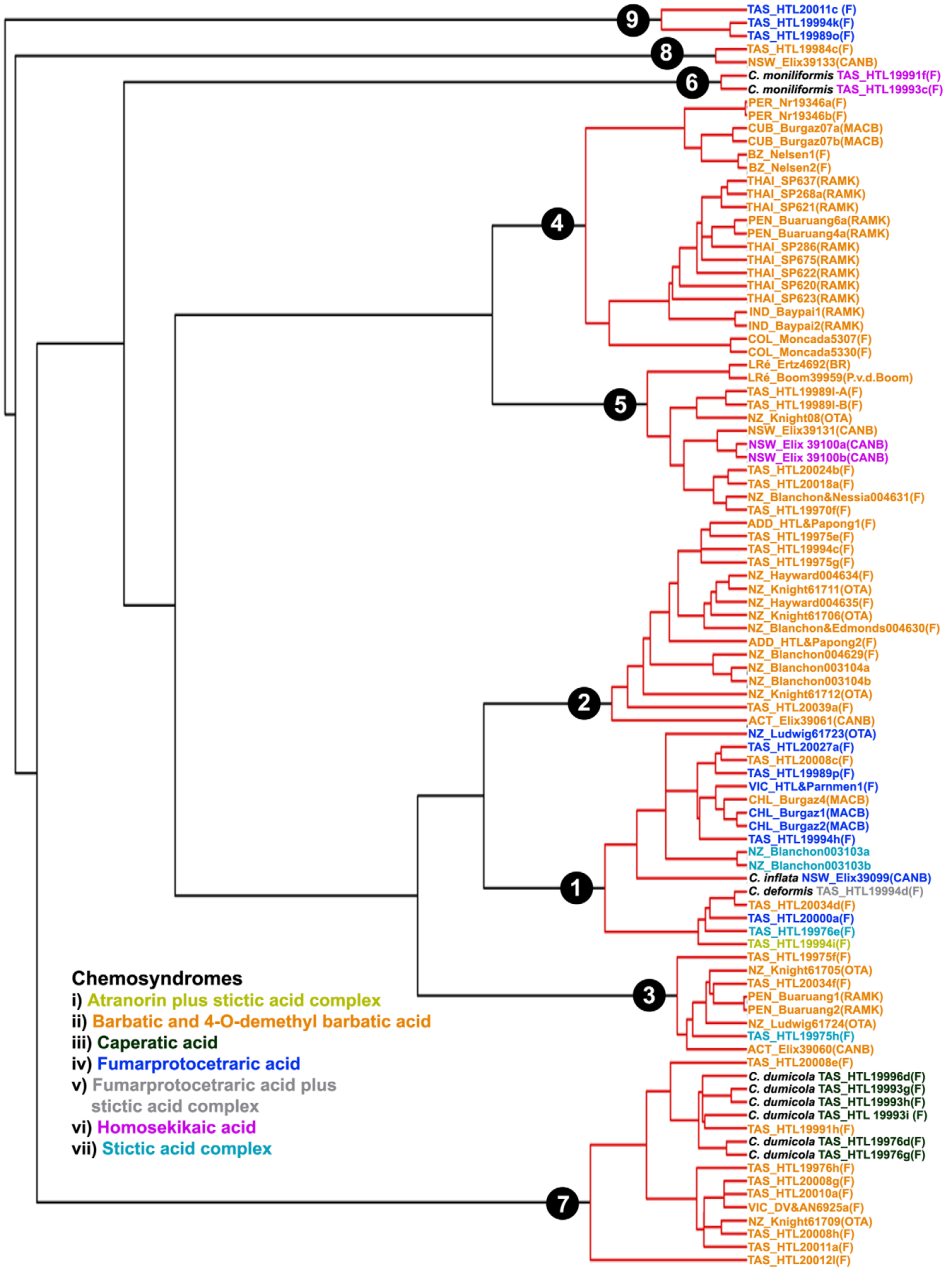
Ultrametric gene genealogy and clusters of specimens recognized as putative species by the single-threshold method of GMYC. Nodes of genetic clusters recognized as putative species are highlighted in red.

In the LTT plot (Fig. 2A) an increase in branching rates has been found at the tips of the linearized trees. In the single-threshold analysis the GMYC model was preferred over the null model of uniform (coalescent) branching rates (*log*L_GMYC_  = −475.368 vs. *log*L_0_ = −463.811; 2ΔL = 23.113, *p*<0.001). Confidence intervals for the estimated number of species ranged from 9 to 11. The model fitted the switch in the branching pattern at −0.202281, leading to an estimate of 9 putative species (Fig. 3).

**Figure 4:**
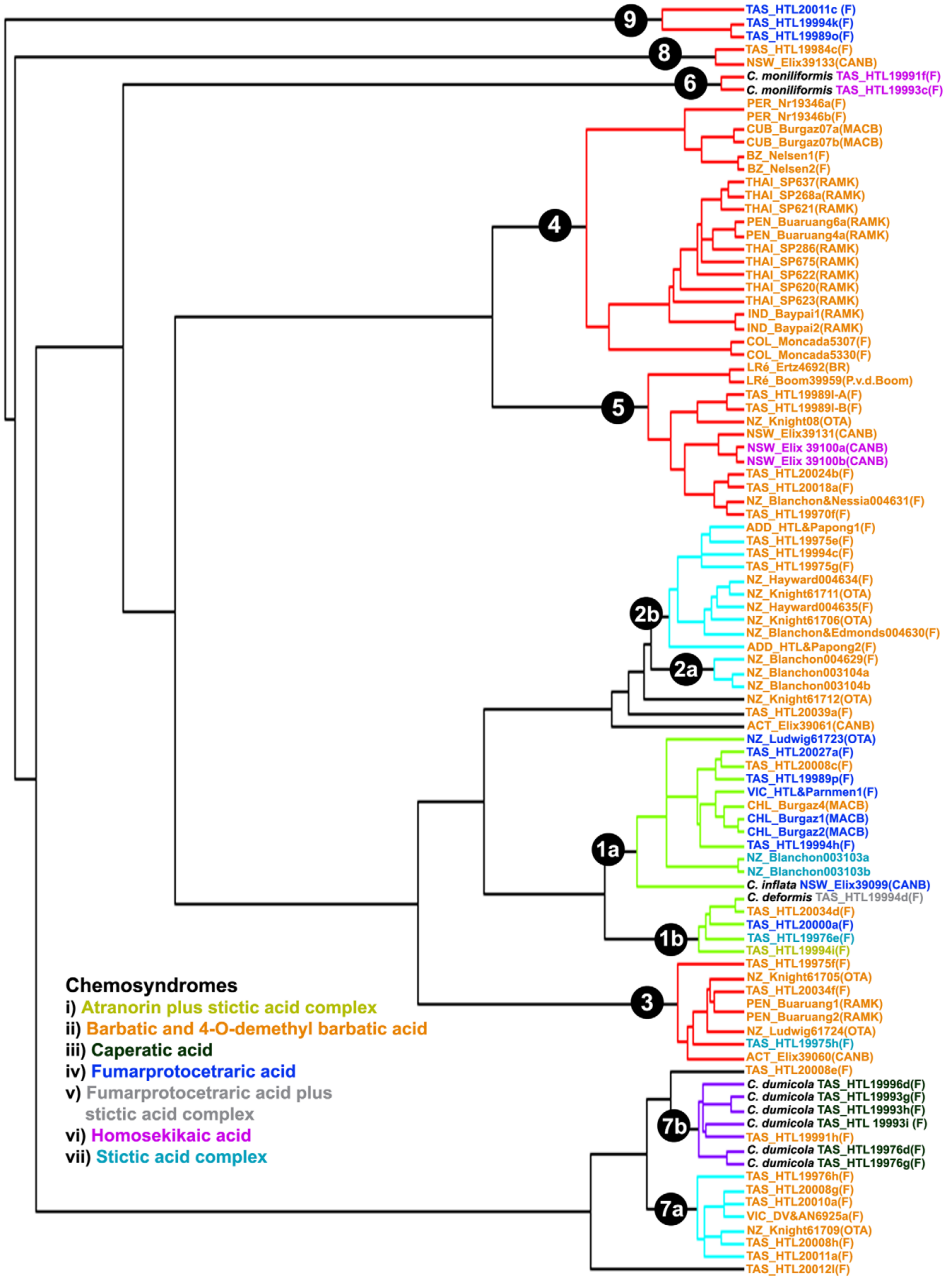
Ultrametric gene genealogy and clusters of specimens recognized as putative species by the multiple-threshold method of GMYC. Nodes of genetic clusters recognized as putative species are highlighted in red, light blue, violet and green.

In the multiple-threshold analysis four switches were identified (Fig. 2B). The GMYC model was preferred over the null model of uniform (coalescent) branching rates (*log*L_GMYC_  = −478.457 vs. *log*L_0_ = −463.811; 2ΔL = 29.291, *p*<0.001). Confidence intervals for the estimated number of species ranged from 8 to 13. The model fitted the switch in the branching pattern at −0.2023, −0.1379, −0.0970, and −0.0602, leading to an estimate of 12 putative species and five single, unclassified individuals (Fig. 4).

The likelihood values of the single and multi-threshold analyses were not significantly different (*p* = 0.722), suggesting that the more complex multi-threshold analysis did not yield a significant improvement of the results. The RaxML tree obtained from a concatenated data set was used to illustrate the delimitation of putative species recognized by the single- and multiple-threshold methods of the GMYC approach (Fig. 1).

### Evaluation of putative species in a genealogical framework

We used a genealogical concordance approach that takes the presence of clades in single-locus genealogies as evidence that these represent distinct lineages as discussed above. The presence of putative species identified by GMYC and their ML bootstrap support values are shown in Table 2 for the single and multiple threshold methods, respectively. Putative species 2 and 6–9 were present in all single-locus analyses, while the putative species 1 and 5 were present in three of the four single-locus phylogenies (Table 2a). Putative species 3 was present in half of the single-locus analyses, while putative species 4 only formed a monophyletic group in the GAPDH analyses. For the clades that were identified additionally in the multi-threshold GMYC analysis, putative species 1a, 2b, 7a, and 7b were present in all single-locus analyses (Tab. 2b), while putative species 1b and 2a were present in all but one single-locus tree.

**Table 2:** Genealogical concordance.

	GMYC	combined	IGS	ITS	GAPDH	Mcm7
Single threshold	1	+	78	−	+	+
	2	100	+	86	81	+
	3	83	+	93	−	−
	4	+	−	−	+	−
	5	+	−	+	+	+
	6	100	99	100	99	86
	7	+	+	92	100	+
	8	100	100	100	97	100
	9	100	100	100	100	100
Multiple threshold	1a	93	+	93	+	+
	1b	99	80	96	−	91
	2a	96	+	+	+	−
	2b	+	+	+	+	+
	3	83	+	93	−	−
	4	+	−	−	+	−
	5	+	−	+	+	+
	6	100	99	100	99	86
	7a	99	95	84	+	+
	7b	86	+	82	+	+
	8	100	100	100	97	100
	9	100	100	100	100	100

Presence of clades and ML bootstrap support values of putative species as suggested by the a) single threshold and b) multiple threshold methods of GMYC (+ = present, but not supported; − = not present).

Single threshold method.

### Species tree analyses

*Beast analyses resulted in a 9-species scenario species tree where clades 1–5 formed a well-supported clade and the sister-group relationship of this clade and clade 7 received significant support, while other relationships in the tree lacked support (Fig. S2A). The 12-species scenario *Beast tree had a similar topology (Fig. S2B), in which clades 1–5 formed a well-supported monophyletic group. Also the sister-group relationships of clades 2a and b and 7a and b each received support.

We used the protocol by Carstens and Dewey [72] to generate species trees from a given set of gene trees under different species delimitation scenarios. Using the same set of gene trees, we compared scenarios that included a 9-species, 12-species scenario, and a 1-species scenario, as explained under Material and Methods. An information-theoretic approach that accommodates for number of parameters strongly supported a 12-species scenario (Table 3) over the set of other species delimitation scenarios. The 1-species classification scenario received the lowest likelihood scores.

**Table 3:** Likelihood scores for STEM analysis of species delimitation scenarios (k =  number of parameters, high Δ lnL means high support for a given scenario).

Scenario	-lnL	k	Δ lnL	Bonferroni corrected *P*
1-species	67176.8169	2	0	
9-species	67127.7905	10	49.0264	<0.001
12-species	65295.6814	13	1881.1355	<0.001

### Association of phenotypical characters with putative species

**Figure 5:**
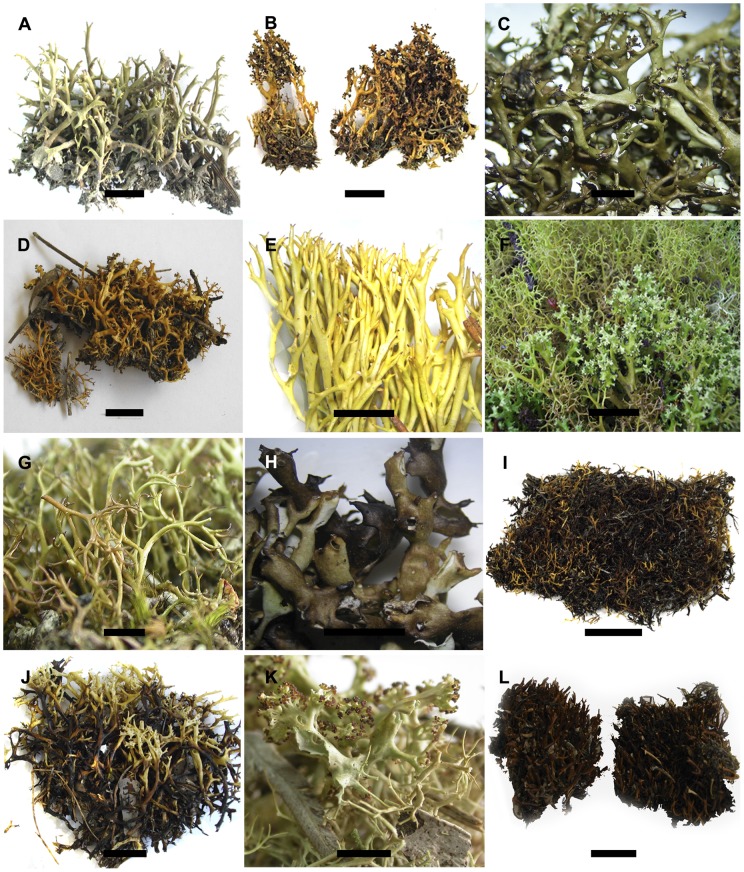
Morphological characteristics of the putative *Cladia* species identified using the multiple threshold method of GMYC. Habits of A)  =  Putative species 1a ( = *C. inflata*, Elix 39099 [CANB]); B)  =  Putative species 1b ( = *C. deformis*, HTL 19994d [F]); C)  =  Putative species 2a (Blanchon 003104a [F]); D)  =  Putative species 2b (HTL 19975e [F]); E)  =  Putative species 3 (HTL 19975f [F]); F)  =  Putative species 4 (SP 286 [RAMK]), G)  =  Putative species 5 (Elix 39100 [CANB]), H) Putative species 6 ( = *C. moniliformis*, HTL 19991h [F]); I)  =  Putative species 7a (HTL 19976h [F]); J)  =  Putative species 7b ( = *C. dumicola*, HTL 19993h [F]); K)  =  Putative species 8 (Elix 39131 [CANB]); L)  =  Putative species 9 (HTL 19989o [F]). Scale bars  = 10 mm.

Morphological and chemical characters and distributional ranges found in the samples belonging to the 9, resp. 12 putative species identified using the two methods of GMYC are listed in Table 4, table S2 and illustrated in Fig. 5. We calculated the homoplasy indices (consistency index CI and retention index RI) for 27 phenotypical characters using the ML tree of the combined data set as reference. A number of characters showed high levels of homoplasy, as indicated by their relatively low CI and/or RI values. Some characters, such as branching of fertile pseudopodetia, matt vs. glossy surface, and length of conidia, which characterize *C. moniliformis* show no homoplasy, since they represent autapomorphies of that species. The results of the contingency table tests for the 9, resp. 12 putative species are listed in Table 4. Characters referring to fertile pseudopodetia were excluded from this analysis, since the sampling number was too low (due to numerous sterile specimens of some clades). We also excluded the three autapomorphic characters of *C. moniliformis* described above. Significant associations of characters with clades in the 9-species scenario were found in 17 characters, including morphology of the sterile pseudopodetia (eight characters) and for nine chemical characters. For the additional four species identified by GMYC using the multi-threshold method, significant associations were found for six chemical characters. Some putative species are characterized by their morphology in combination with their secondary chemistry, while others cannot be distinguished from others based on morphology or chemistry alone. Table 5 lists the character states found in the putative species of characters that were found to have phylogenetic signal in the contingency table tests, and additionally characters of the fertile pseudopodetia when found. In addition the known geographical distribution of each putative species is indicated.

**Table 4:** Homoplasy measures of phenotypical characters and their association with phylogenetic structure in the *Cladia aggregata* complex.

Morphs	No.	Characters states	CI	RI	Results of contingency table tests (*p-*value)	
					9 spp.	12 spp.*
Sterile pseudopodetia	1	Sterile pseudopodetia not inflated (0), Inflated (1), Inflated with bulbous or cylindrical segment (2)	0.09	0.53	<0.001 (df = 16)	0.066 (df = 4)
	2	Sterile pseudopodetia dichotomously branched (0), irregularly branched (1)	0.25	0.40	<0.001 (df = 8)	-
	3	Sterile pseudopodetia repeatedly branched (0), unbranched or with occasional branches (1)	0.05	0.29	<0.001 (df = 8)	0.161 (df = 4)
	4	Surface glossy (0), matt (1)	0.25	0.25	<0.001 (df = 8)	0.409 (df = 4)
	5	Surface color pale yellow-green (0), yellow to olive brown (1), reddish brown (2), chestnut brown to blackish brown (3),	0.06	0.28	<0.001 (df = 24)	0.127 (df = 12)
	6	Axils not perforate (0), perforate (1)	0.04	0.53	<0.001 (df = 8)	0.165 (df = 4)
	7	Apices mostly tapered (0), rounded and blunt (1), acute (2), truncate (3)	0.09	0.38	<0.001 (df = 24)	0.088 (df = 8)
	8	Perforation absent to rare (0), abundant (1)	0.05	0.58	<0.001 (df = 8)	0.347 (df = 4)
Fertile pseudopodetia	9	Fertile pseudopodetia shorter than sterile pseudopodetia (0) more robust and taller that sterile pseudopodetia (1) more slender and taller that sterile pseudopodetia (2)	0.20	0.42		
	10	Cylindrical fertile pseudopodetia (0), inflated (1), inflated with bulbous or cylindrical segment (2)	0.20	0.33		
	11	Fertile pseudopodetia dichotomously branched (0), irregularly branched (1)	1	1		
	12	Branching racemose (0), corymbose (1)	0.25	0.50		
	13	Surface glossy (0), matt (1)	1	1		
	14	Surface color pale yellow-green (0), yellow to olive brown (1), reddish brown (2), chestnut brown to blackish brown (3),	0.25	0.43		
	15	Axils not perforate (0), axils perforate (1)	0.20	0.20		
	16	Perforation absent to rare (0), abundant (1)	0.14	0.33		
	17	Conidia <8 µm long (0), >8 µm long (1)	1	1		
Secondary chemistry	18	Beta-orcinol p-depsides absent (0), present (1)	0.06	0.61	<0.001 (df = 8)	<0.001 (df = 4)
	19	Atranorin absent (0), present (1)	0.50	0	0.796 (df = 8)	0.227 (df = 4)
	20	Barbatic acid absent (0), present (1)	0.06	0.65	<0.001 (df = 8)	<0.001 (df = 4)
	21	Beta-orcinol depsidones absent (0), present (1)	0.08	0.65	<0.001 (df = 8)	<0.001 (df = 4)
	22	Fumarprotocetraric acid absent (0), present (1)	0.09	0.52	<0.001 (df = 8)	0.021 (df = 4)
	23	Norstictic acid absent (0), present (1)	0.12	0.41	<0.001 m(df = 8)	<0.001 (df = 4)
	24	Stictic acid absent (0), present (1)	0.12	0.41	<0.001 (df = 8)	<0.001 (df = 4)
	25	Orcinol m-depsides absent (0), present (1)	0.50	0.66	<0.001 (df = 8)	-
	26	Homosekikaic acid absent (0), present (1)	0.50	0.66	<0.001 (df = 8)	-
	27	Caperatic acid absent(0), present (1)	0.50	0.85	<0.001 (df = 8)	<0.001 (df = 4)

Homoplasy measured using the consistency index (CI) and retention index (RI). Low values of these point toward homoplasy. The morphologically deviating *C. moniliformis* was excluded from the contingency table tests (*Only additional putative species tested for the 12-species scenario).

**Table 5:** Phenotypic characteristics of the putative species focusing on characters shown to have significant association with clades in contingency table test (Tab. 4), plus characters of fertile pseudopodetia, secondary chemistry, and geographical origin of samples.

Putative species	Morphological characteristics	Chemosyndromes	Geographical origins
1	Sterile pseudopodetia are inflated to evenly tapered to cylindrical, rarely with perforation, the color varied from pale yellow to chestnut-brown. Fertile pseudopodetia are slender and taller than sterile ones, mostly corymbosely branched towards the apex.	i) atranorin plus stictic acid complex; ii) barbatic acid; iii) fumarprotocetraric acid; iv) fumarprotocetraric acid plus stictic acid complex & v) stictic acid complex chemosyndromes	Australia, New Zealand and southern South America
1a	Sterile pseudopodetia are inflated to evenly cylindrical, rarely with perforation, the color varied from pale yellow to grayish, and the specimens are fragile when dry.	i) barbatic acid; ii) fumarprotocetraric acid & iii) stictic acid complex chemosyndromes	Australia, New Zealand and southern South America
1b	Sterile pseudopodetia are erect and tapered, pale yellow to chestnut-brown and glossy, mostly dichotomously branched, and sparsely perforated. Fertile pseudopodetia are slender and taller than sterile ones, mostly corymbosely branched towards the apex	i) atranorin plus stictic acid complex; ii) barbatic acid; iii) fumarprotocetraric acid; iv) fumarprotocetraric acid plus stictic acid complex & v) stictic acid complex chemosyndromes	Australia
2	Sterile pseudopodetia are inflated and tapered, glossy, pale yellow to brownish, and mostly dichotomously branched toward the apex. Perforations are rare. Fertile pseudopodetia are robust and either inflated or not, and racemosely branched.	Barbatic acid chemosyndrome	Australia and New Zealand
2a	Sterile pseudopodetia are mostly inflated, glossy, pale yellow-green, and mostly dichotomously branched toward the apex. Perforations are rare.	Barbatic acid chemosyndrome	New Zealand
2b	Sterile pseudopodetia are inflated and tapered, glossy, pale yellow to brownish, and mostly dichotomously branched toward the apex. Perforations are rare. Fertile pseudopodetia are robust and either inflated or not, and racemosely branched. Perforations are abundant.	Barbatic acid chemosyndrome	Australia and New Zealand
3	Sterile pseudopodetia are inflated, glossy, yellow to brownish, and mostly dichotomously branched toward the apex. Perforations are abundant. The sizes fertile pseudopodetia are extremely variable, with yellow to brownish yellow surfaces and racemosely branched.	Barbatic acid and stictic acid chemosyndromes	Australia and Southeast Asia
4	Sterile pseudopodetia are extremely variable in surface color and size ranging from 1 to 10 cm, usually richly perforated and richly branched, pale to dark or reddish-brown or pale yellow or pale green to greenish. The fertile pseudopodetia are thicker and racemosely branched toward the apex.	Barbatic acid chemosyndrome	Neotropics, India and Southeast Asia
5	Sterile pseudopodetia are not inflated and glossy, with a chestnut-brown to greenish surface. The fertile pseudopodetia are shorter than sterile pseudopodetia and racemosely branched toward the apex.	Homosekikaic acid and barbatic acid chemosyndromes	Australia, La Réunion and New Zealand
6	Sterile pseudopodetia are inflated with bulbous segments, smooth, yellow brown to blackish, and sparsely perforated. Fertile pseudopodetia are more slender with racemosely branched at the apex.	Homosekikaic acid chemosyndrome	Tasmania
7	Sterile pseudopodetia are tapered, (1)2–3(5) cm tall, not perforated, glossy and smooth, pale to brownish or dark. Fertile pseudopodetia are robust, flattened, not constricted at the base, 3–5 cm tall, perforated, and racemosely branched.	barbatic acid and caperatic acid chemosyndromes	Australia and New Zealand
7a	Sterile pseudopodetia are tapered, not perforated, glossy and smooth, pale yellow-green to brownish or dark. Fertile pseudopodetia are slender, perforated, and racemosely branched.	barbatic acid chemosyndrome	Australia and New Zealand
7b	Sterile pseudopodetia are tapered, not perforated, glossy and smooth, pale to brownish or dark. Fertile pseudopodetia are robust, flattened, not constricted at the base, perforated, and racemosely branched.	barbatic acid and caperatic acid chemosyndromes	Tasmania
8	Sterile pseudopodetia are tapered, not perforated, glossy and smooth, pale yellow green. Fertile pseudopodetia are shorter that sterile morph, perforated, and racemosely branched.	barbatic acid chemosyndrome	Australia
9	Sterile pseudopodetia are swollen, areolate, 3–6 cm tall, glossy and usually smooth, chestnut-brown to blackish brown, not perforated, and di- or trichotomously branched. Fertile pseudopodetia are robust and taller than sterile pseudopodetia, necrotic at the base, racemosely branched.	Fumarprotocetraric acid chemosyndrome	Tasmania

## Discussion

This study is another example for the hidden diversity in lichen-forming fungi challenging the phenotypically-based species circumscription in these organisms, which underestimates the true species diversity [38]. We also found considerable spatial structure in the data sets with no haplotype occurring in more than one geographic region. This is remarkable for lichenized fungi, where most studies revealed wide distributions of single haplotypes [26], [73]–[75] and less geographic structure. Several putative species have restricted geographical distributions (Table 5), such as endemic to mainland Australia (putative species 1b, 8), Tasmania (putative species 6, 7b, 9), or New Zealand (putative species 2a) or have Australasian or austral distributions (putative species 1a, 2b, 3, 7a), while only two putative species have broad intercontinental distributions. These include putative species 4 with samples from the Neotropics, India and Southeast Asia. Additional sampling, however, is required to test whether the Asian material, which forms a monophyletic and well-supported clade (Fig. 1) is indeed conspecific with the Neotropical specimens, which are paraphyletic. Further, putative species 4 is not supported, indicating uncertainty in the circumscription of this species. Putative species 5 also lacks support and the two samples from Reunion form a well-supported clade which is a sister-group to the Australasian samples, which also form a well-supported monophyletic clade (Fig. 1). To address the question whether putative species 5 consists of more than one species will require further studies with an extended sampling from Africa.

The *Cladia aggregata* complex is a controversial group with no obvious correlation of morphological and chemical variation. Some morphological and/or chemical variants have previously been recognized as distinct species. These include *Cladia inflata* with inflated pseudopodetia [76] and *C. moniliformis* with inflated pseudopodetia with bulbous segments [53], while others were predominantly recognized based on their chemistries [5]. Another morphologically deviating population was described as *C. globosa* from the Neotropics, but could not be included in this study since no fresh collections were available [52]. None of the recently described taxa have been tested using molecular data so far. Here we have most of the species currently recognized included in the molecular study, except *C. globosa, C. mutabilis* and *C. oreophila*. We have been able to include material of most chemotypes described in the *C. aggregata* complex.

The previously described species *Cladia deformis, C. inflata* and *C. dumicola* [5], [76] were not supported in their current circumscription by our analyses. In fact, the previously recognized species in the *C. aggregata* group, except *C. inflata* and *C. moniliformis* (Fig. 1), did not form distinct clades. The first previously recognized species, *Cladia inflata* formed a strongly supported clade which was nested within putative species 1a (Fig. 3). However, since it forms a sister-group to the remaining specimens of putative species 1a, additional sampling is necessary to identify the taxonomic status of this species. The samples in this clade were all collected in Australia, New Zealand and southern South America. *Cladia deformis* clustered within putative species 1b and nested among four different chemotypes of *C. aggregata* (Fig. 1). *Cladia dumicola*, which contains caperatic acid, was intermixed with *C. aggregata* containing the barbatic acid chemosyndrome (Fig. 3) forming putative species 7b.

The use of secondary metabolic characters for delimiting species in lichenized fungi has been supported by some phylogenetic studies (e.g. [30], [31], [77]–[80], while other studies did not find a correlation between chemotypes and lineages found in molecular phylogenetic analyses [81]–[86]. There is a phylogenetic pattern in the distribution of secondary metabolites among putative species within the *Cladia aggregata* complex. However, it appears that chemical characters cannot consistently be used to characterize species-level lineages. For example, the barbatic acid chemosyndrome occurred in almost all clades (Fig. 3). Hence, the presence of chemosyndromes alone does not indicate the affiliation of a sample to a lineage in these fungi. Individuals within each of the chemotypes were morphologically variable and individuals in different chemotypes often shared each of morphological traits (Fig. 1). This could be due to the fact that some chemical and morphological characteristics, such as branching pattern or surface colors are homoplasious. The absence of a correlation of some phenotypical characters and phylogenetic placement may be due to different reasons. It seems obvious that they share ancestral polymorphisms and might retain some features of their ancestor's morphology. Moreover, our understanding on the effect of ecological conditions on morphological and chemical variation is still poor. Our study adds another example of our lack of knowledge in understanding adaptive values of phenotypical characters.

The GMYC method has previously been applied to species delimitation in various groups of organisms, such as insects [63], skinks [87], amphibians [88], rodents [71] and rotifers [89]. The single-threshold model identified fewer putative species (9) than the multiple-threshold model (12) in our analysis. Since the likelihood values of the single- and multi-threshold analysis were not significantly different, we employed two additional approaches: 1) a genealogical concordance approach comparing the presence of putative species in single-locus analyses and 2) a coalescent-based approach that uses a species tree estimation method to choose among species delimitation scenarios. Genealogical concordance among different loci provides strong evidence that clades represent reproductively isolated lineages [57]–[59]. However, different loci may give conflicting or ambiguous results due to various evolutionary processes associated with speciation (e.g., incomplete lineage sorting). Thus, using multi-locus sequence data and multiple empirical methods allow us to establish more robust species boundaries [38]. The four additional species identified by the multiple threshold method of GMYC were present in the majority of single-locus analyses and hence support that our data set includes 12 putative species. The coalescent-based species tree method significantly preferred the 12-species scenario. Consequently, we propose that the sampled specimens of the *Cladia aggregata* complex belong to 12 distinct species.

While some clades, such as putative species 6 (*C. moniliformis*), are characterized by several characters, other clades can be phenotypically characterized by combination of characters only or lack obvious phenotypical differences to other putative species. Our morphometric analyses demonstrated that several morphological and chemical characters were associated with clades. Since these characters can only be used in combination and to some extent to characterize clades, molecular data are necessary in most cases to unequivocally assign specimens to putative species. Despite the limitations in circumscribing species using molecular data, the effective use of genetic data appears to be essential to appropriately and practically identify natural groups in some phenotypically cryptic lichen-forming fungal lineages [33], [37], [80], [90]–[93]. Our re-examination also identified morphological and chemical characters that characterize, at least in combination, some of the putative species as also found in other groups of lichenized fungi [25], [26], [36], [90], [92], [94]. Under the general lineage species concept [95], [96], more independent properties associated with putative species boundaries are associated with a higher degree of corroboration, resulting in a truly integrative approach to species discovery. Hence the association of phenotypical characters with at least some of the putative species gives us confidence that the clades found in our analyses indeed represent distinct lineages, i.e. species.

## Materials and Methods

### Taxon sampling

The taxon sampling included most morpho- and chemotypes known from the *Cladia aggregata* group and two samples of *C. schizopora* as outgroup based on previous molecular studies [7], [8]. In total over 150 samples were studied, but here only 126 samples are included for which we obtained sequences of at least three of the four loci studied. In the current classification these belong to 6 species. Loci sequenced were nuITS rDNA, nuIGS rDNA and the protein-coding GAPDH and Mcm7 genes. Specimens and sequences used for the molecular analyses are listed in table S1.

### Molecular methods

Total DNA was extracted from herbarium material using the DNeasy Plant Mini Kit (Qiagen) following the instructions of the manufacturer. Dilutions (10^−1^ up to 10^−2^) of DNA were used for PCR amplifications. Four gene regions were used for the study: i) the intergenic spacer (IGS) region of the nuclear ribosomal DNA, ii) the internal transcribed spacer (ITS) region of the nuclear ribosomal DNA, and two nuclear protein-coding genes: glyceraldehyde-3-phosphate dehydrogenase (GAPDH) and DNA replication licensing factor Mcm7. Primers for PCR amplification were i) IGS regions: IGS-12a and NS1R [97], [98]; ii) ITS regions: ITS1F-5′ [99] and ITS4-3′ [100]; iii) the protein-coding GAPDH gene: Gpd1-LM and Gpd2-LM [101] and iv) the protein-coding Mcm7 gene: Mcm7-709for and Mcm7-1447rev [102]. In addition, four newly designed primer pair for the protein-coding GAPDH and Mcm7 genes were designed for this study: Gpd-F124 (5′ AGC CTC ACT ATG CAG TAC GCT CTT 3′) and Gpd-R922 (5′ TTG TCT CCG TTC AAG TCG CT 3′), *Cladia*Mcm7-F (5′AAG CAG TTC ACC CCA TTG AC 3′) and *Cladia*Mcm7-R (5′ CCC ATT TCT TTC GTG ACA CC 3′).

Thermal cycling parameters were: initial denaturation for 5 min at 94°C, followed by 35 cycles of 30 s –1 min at 95°C, 30–45 s at 52–55°C, 1–1.30 min at 72°C, and a final elongation for 10 min at 72°C. The cycle sequencing conditions was as described previously [8].

Sequence alignments were done separately for each data set using BioEdit [103]. Ambiguous regions in the nuITS alignment were removed manually before analysis.

### Chemistry

The chemical constituents were identified using thin layer chromatography (TLC) [104], [105] and gradient-elution high performance liquid chromatography (HPLC) [106].

### Phylogenetic analyses

To test for potential conflict, maximum likelihood bootstrap analyses were performed on each individual data set with the same settings as in the concatenated analysis and with models as shown in Table 1, and 75% bootstrap consensus trees were examined for conflict [107]. Since no conflicts (i.e. well supported differences in the topology) were found, multi-gene data sets were analyzed under Bayesian approach (B/MCMC) and maximum likelihood (ML).

A Bayesian analysis was performed using MrBayes 3.1.2 [108] with substitution models as shown in Table 1. The data set were partitioned into eight parts including ITS, IGS and the three codon positions of each of the protein-coding GAPDH and Mcm7. Each partition was allowed to have own parameter values [109]. No molecular clock was assumed. Heating of chains was set to 0.2. Posterior probabilities were approximated by sampling trees using a variant of Markov Chain Monte Carlo (MCMC) method. Number of generations was 10 million. To avoid autocorrelation, only every 1000th tree was sampled. The first 40,000 generations were discarded as burn in. We plotted the log-likelihood scores of sample points against generation time using TRACER v1.4.1 (Tracer website. Available: http://tree.bio.ed.ac.uk/software/tracer/. Accessed 2012 Nov 19) to ensure that stationarity was achieved after the first 40,000 generations by checking whether the log-likelihood values of the sample points reached a stable equilibrium value [108]. Additionally, we used AWTY [110] to compare split frequencies in the different runs and to plot cumulative split frequencies to ensure that stationarity was reached. Of the remaining 19,920 trees (9960 from each of the parallel runs) a majority rule consensus tree with average branch lengths was calculated using the sumt option of MrBayes. Posterior probabilities were obtained for each clade.

A maximum likelihood (ML) analysis was performed for each locus and the combined data set in RAxML 7.2.6 [111] using the GTRGAMMA model with 25 rate parameter categories. Support was then estimated by performing 1000 bootstrap pseudoreplicates [112]. Only clades with bootstrap support equal or above 70% under ML and posterior probabilities ≥0.95 in the Bayesian analysis were considered as strongly supported. Phylogenetic trees were visualized using the program Treeview [113].

### General Mixed Yule Coalescent (GMYC) Species Delimitation

We used the DNA-based approach by Pons *et al*. [62] to delimit species. This method aims at detecting shifts in branching rates between intra- and interspecific relationships. Within a likelihood framework it uses chronograms to compare two models: a) a null model under which the whole sample derives from a single population obeying a coalescent process and b) an alternative general mixed Yule coalescent (GMYC) model. The latter combines equations that separately describe branching patterns within and among distinct lineages. The latter follow a Yule model including speciation and extinction, whereas intraspecific relationships follow a coalescent process. A likelihood ratio test (LRT) is used to evaluate whether the null model can be significantly rejected. If the GMYC model fits the data significantly better than the null model, the threshold T allows to estimating the number of species present in the data set.

The ML tree obtained from a RAxML search using the combined data set was used for the analysis. The two outgroup samples (*C. schizopora*) were excluded from the data set using the drop.tip command in ape [114]. A chronogram was calculated from the ML tree using the penalized likelihood method [115] as implemented in the chronopl command in ape. The GMYC method requires a fully dichotomous chronogram and thus we used multdivtime [116] to convert our chronogram into a fully dichotomous chronogram with internal branches of length zero, where appropriate. This modified chronogram was then analyzed using the gmyc package in SPLITS in R (version 2.10, www.cran.r-project.org), using the single and multiple threshold methods. After optimization, we plotted the lineage through time (LTT) plot [117] with the threshold indicated and a chronogram that had the putative species indicated. Finally, we used the summary command to summarize the output statistics, including the results of the LRT and the indication of the numbers of clusters and entities.

### Species Tree analyses

Using *Beast [118], we generated species trees for two species delimitation scenarios: the 9-species scenario and the 12-species scenario, as described below. For these analyses we used all loci sampled that were also used in the concatenated analyses described above. Sixty million generations with a burn-in of 20 million were run and trees combined using the program LogCombiner in the Beast software package [119].

We applied a coalescent-based approach to test alternative hypotheses of species delimitation. We used the program STEM [120] following the protocol outlined in Carstens and Dewey [72] to estimate likelihood scores of alternative species delimitation scenarios among species trees. We performed a set of analyses assuming a classification including all samples in the complex in 1 species (1-species scenario), distinction of nine clades as species as suggested by the single threshold method of GMYC (9-species scenario), and accepting 12 clades as distinct species, as suggested by the multiple threshold method of GMYC (12-species scenario). Species tree analyses were performed with maximum likelihood gene trees estimated for each locus separately RAxML v7.2.7 [111], [121]. STEM requires fully resolved gene trees, thus polytomies were resolved using MULTI2DI in the ape package [122]. ML scores were evaluated using likelihood-ratio tests (LRTs) to assess statistical significance after correcting for multiple comparisons with a Bonferroni correction.

### Association of phenotypical characters with putative species

We used Mesquite [123] to calculate the homoplasy indices (consistency index CI and retention index RI) for 27 phenotypical characters using the ML tree of the combined data set as reference. Contingency table tests were done using the tools available on the in-silico website (Available: http://in-silico.net/statistics/. Accessed 2012 Nov 19). For this analysis, autapomorphic characters of *C. moniliformis* were excluded and also traits of fertile pseudopodetia, since the sampling number was too low for those characters.

## Supporting Information

Figure S1 **Majority-consensus tree depicting relationships within the** ***Cladia aggregata*** **complex.** A) ITS rDNA, B) IGS rDNA, C) protein-coding GAPDH and D) protein-coding Mcm7 sequences.
(DOC)

Figure S2 **Species trees of the** ***Cladia aggregata*** species complex inferred from four loci using *Beast. Posterior probabilities are indicated next to each node. All individuals were assigned a priori to either nine species (A) or 12 species (B).
(DOC)

Table S1 **Specimens used in this study.** New sequences are indicated in bold.
(DOC)

Table S2 **A data matrix containing morphological and chemical characters of the genus** ***Cladia*** **under study.**
(DOC)

## References

[1] Wiens JJ, Penkrot TA (2002) Delimiting species using DNA and morphological variation and discordant species limits in spiny lizards (*Sceloporus*). Systematic Biology 51: 69–91. 11943093 10.1080/106351502753475880

[2] Cracraft J (1983) Species concepts and speciation analysis. Current Ornithology 1: 159–187.

[3] Mayr E (1963) Animal species and evolution. Cambridge, MA: Harvard University Press.

[4] Purvis OW (1997) The species concept in lichens. In: Claridge MF, Dawah HA, Wilson MRe, editors. Species: The Units of Biodiversity: The Systematics Association Special Volume Series, Chapman & Hall, London. 109–134.

[5] Kantvilas G, Elix JA (1999) Studies on the lichen genus *Cladia* Nyl. in Tasmania: the *C. aggregata* complex. Muelleria 12: 135–162.

[6] Culberson WL, Culberson CF, Johnson A (1983) Genetic and environmental effects on growth and production of secondary compounds in *Cladonia cristatella* . Biochemical Systematics and Ecology 11: 77–84.

[7] Lumbsch HT, Parnmen S, Rangsiruji A, Elix JA (2010) Phenotypic disparity and adaptive radiation in the genus *Cladia* (Lecanorales, Ascomycota). Australian Systematic Botany 23: 239–247.

[8] Parnmen S, Rangsiruji A, Mongkolsuk P, Boonpragob K, Elix JA, et al (2010) Morphological disparity in Cladoniaceae: The foliose genus *Heterodea* evolved from fruticose *Cladia* species (Lecanorales, lichenized Ascomycota). Taxon 59: 841–849.

[9] Stenroos SK, DePriest PT (1998) SSU rDNA phylogeny of cladoniiform lichens. American Journal of Botany 85: 1548–1559. 21680313

[10] Wedin M, Döring H (1999) The phylogenetic relationship of the Sphaerophoraceae, *Austropeltum* and *Neophyllis* (lichenized Ascomycota) inferred by SSU rDNA sequences. Mycological Research 103: 1131–1137.

[11] Tehler A, Irestedt M (2007) Parallel evolution of lichen growth forms in the family Roccellaceae (Arthoniales, Ascomycota). Cladistics 23: 432–454.

[12] Högnabba F (2006) Molecular phylogeny of the genus *Stereocaulon* (Stereocaulaceae, lichenized ascomycetes). Mycological Research 110: 1080–1092. 16934965 10.1016/j.mycres.2006.04.013

[13] Blanco O, Crespo A, Elix JA, Hawksworth DL, Lumbsch HT (2004) A molecular phylogeny and a new classification of parmelioid lichens containing *Xanthoparmelia*-type lichenan (Ascomycota: Lecanorales). Taxon 53: 959–975.

[14] Crespo A, Lumbsch HT (2010) Cryptic species in lichen-forming fungi. IMA Fungus 1: 167–170. 22679576 10.5598/imafungus.2010.01.02.09 PMC3348775

[15] Kroken S, Taylor JW (2001) A gene genealogical approach to recognize phylogenetic species boundaries in the lichenized fungus Letharia. Mycologia 93: 38–53.

[16] Hodkinson BP, Lendemer JC (2011) Molecular analyses reveal semi-cryptic species in *Xanthoparmelia tasmanica* . Bibliotheca Lichenologica 106: 108–119.

[17] Vondrak J, Riha P, Arup U, Søchting U (2009) The taxonomy of the *Caloplaca citrina* group (Teloschistaceae) in the Black Sea region; with contributions to the cryptic species concept in lichenology. Lichenologist 41: 571–604.

[18] Crespo A, Perez-Ortega S (2009) Cryptic species and species pairs in lichens: A discussion on the relationship between molecular phylogenies and morphological characters. Anales del Jardín Botánico de Madrid 66: 71–81.

[19] Wedin M, Westberg M, Crewe AT, Tehler A, Purvis OW (2009) Species delimitation and evolution of metal bioaccumulation in the lichenized *Acarospora smaragdula* (Ascomycota, Fungi) complex. Cladistics 25: 161–172. 10.1111/j.1096-0031.2009.00240.x 34879601

[20] Lumbsch HT, Mangold A, Martin MP, Elix JA (2008) Species recognition and phylogeny of *Thelotrema* species in Australia (Ostropales, Ascomycota). Australian Systematic Botany 21: 217–227.

[21] Ruprecht U, Lumbsch HT, Brunauer G, Green TGA, Türk R (2010) Diversity of *Lecidea* (Lecideaceae, Ascomycota) species revealed by molecular data and morphological characters. Antarctic Science 22: 727–741.

[22] McDonald T, Miadlikowska J, Lutzoni F (2003) The lichen genus *Sticta* in the Great Smoky Mountains: A phylogenetic study of morphological, chemical, and molecular data. Bryologist 106: 61–79.

[23] Molina MD, Crespo A, Blanco O, Lumbsch HT, Hawksworth DL (2004) Phylogenetic relationships and species concepts in *Parmelia* s.str. (Parmeliaceae) inferred from nuclear ITS rDNA and beta-tubulin sequences. Lichenologist 36: 37–54.

[24] Divakar PK, Blanco O, Hawksworth DL, Crespo A (2005) Molecular phylogenetic studies on the *Parmotrema reticulatum* (syn. *Rimelia reticulata*) complex, including the confirmation of *P. pseudoreticulatum* as a distinct species. Lichenologist 37: 55–65.

[25] Divakar PK, Molina MC, Lumbsch HT, Crespo A (2005) *Parmelia barrenoae*, a new lichen species related to *Parmelia sulcata* (Parmeliaceae) based on molecular and morphological data. Lichenologist 37: 37–46.

[26] Wirtz N, Printzen C, Lumbsch HT (2008) The delimitation of Antarctic and bipolar species of neuropogonoid *Usnea* (Ascomycota, Lecanorales): a cohesion approach of species recognition for the *Usnea perpusilla* complex. Mycological Research 112: 472–484. 18314319 10.1016/j.mycres.2007.05.006

[27] McCune B, Schoch C (2009) *Hypogymnia minilobata* (Parmeliaceae), a new lichen from coastal California. Bryologist 112: 94–100.

[28] Goffinet B, Miadlikowska J, Goward T (2003) Phylogenetic inferences based on nrDNA sequences support five morphospecies within the *Peltigera didactyla* complex (lichenized ascomycota). Bryologist 106: 349–364.

[29] Cubero OF, Crespo A, Esslinger TL, Lumbsch HT (2004) Molecular phylogeny of the genus *Physconia* (Ascomycota, Lecanorales) inferred from a Bayesian analysis of nuclear ITS rDNA sequences. Mycological Research 108: 498–505. 15230002 10.1017/s095375620400975x

[30] Lücking R, del Prado R, Lumbsch HT, Will-Wolf S, Aptroot A, et al (2008) Phylogenetic patterns of morphological and chemical characters and reproductive mode in the *Heterodermia obscurata* group in Costa Rica (Ascomycota, Physciaceae). Systematics and Biodiversity 6: 31–41.

[31] Elix JA, Corush J, Lumbsch HT (2009) Triterpene chemosyndromes and subtle morphological characters characterise lineages in the *Physcia aipolia* group in Australia (Ascomycota). Systematics and Biodiversity 7: 479–487.

[32] Högnabba F, Wedin M (2003) Molecular phylogeny of the *Sphaerophorus globosus* species complex. Cladistics 19: 224–232.

[33] Divakar PK, Figueras G, Hladun NL, Crespo A (2010) Molecular phylogenetic studies reveal an undescribed species within the North American concept of *Melanelixia glabra* (Parmeliaceae). Fungal Diversity 42: 47–55.

[34] Thell A, Elix JA, Søchting U (2009) *Xanthoparmelia lineola* s. l. in Australia and North America. Bibliotheca Lichenologica 99: 393–404.

[35] Otálora MAG, Martínez I, Aragón G, Molina MC (2010) Phylogeography and divergence date estimates of a lichen species complex with a disjunct distribution pattern. American Journal of Botany 97: 216–223. 21622381 10.3732/ajb.0900064

[36] Arguello A, Del Prado R, Cubas P, Crespo A (2007) *Parmelina quercina* (Parmeliaceae, Lecanorales) includes four phylogenetically supported morphospecies. Biological Journal of the Linnean Society 91: 455–467.

[37] Leavitt SD, Fankhauser JD, Leavitt DH, Porter LD, Johnson LA, et al (2011) Complex patterns of speciation in cosmopolitan “rock posy” lichens – Discovering and delimiting cryptic fungal species in the lichen-forming *Rhizoplaca melanophthalma* species-complex (Lecanoraceae, Ascomycota). Molecular Phylogenetics and Evolution 59: 587–602. 21443956 10.1016/j.ympev.2011.03.020

[38] Lumbsch HT, Leavitt SD (2011) Goodbye morphology? A paradigm shift in the delimitation of species in lichenized fungi Fungal Diversity 50: 59–72.

[39] Spribille T, Klug B, Mayrhofer H (2011) A phylogenetic analysis of the boreal lichen *Mycoblastus sanguinarius* (Mycoblastaceae, lichenized Ascomycota) reveals cryptic clades correlated with fatty acid profiles. Molecular Phylogenetics and Evolution 59: 603–614. 21443957 10.1016/j.ympev.2011.03.021 PMC3093615

[40] Filson RB (1981) A revision of the lichen genus *Cladia* Nyl. Journal of the Hattori Botanical Laboratory 49: 1–75.

[41] Kantvilas G (1995) Alpine lichens of Tasmania's South West wilderness. Lichenologist 27: 433–449.

[42] Lumbsch HT, Huhndorf SH (2007) Outline of Ascomycota – 2007. Myconet 13: 1–58.

[43] Jahns HM (1970) Untersuchungen zur Entwicklungsgeschichte der Cladoniaceen mit besonderer Berücksichtigung des Podetien-Problems. Nova Hedwigia 20: 1-vi + 1–177.

[44] Ahti T (1982) The morphological interpretation of cladoniiform thalli in lichens. Lichenologist 14: 105–113.

[45] Jahns M, Sensen M, Ott S (1995) Significance of developmental structures in lichens, especially in the genus *Cladonia* . Annales Botanici Fennici 32: 35–48.

[46] Lamb IM, Weber WA, Jahns HM, Huneck S (1972) *Calathaspis*, a new genus of the lichen family Cladoniaceae. Occasional Papers of the Farlow Herbarium of Cryptogamic Botany, Harvard University 4: 1–12.

[47] Jahns HM, Van der Knapp F (1973) Die Flechtengattung *Heterodea* Nyl. Systematik und Ontogenie der Fruchtkörper. Herzogia 2: 437–451.

[48] Filson RB (1978) A revision of the genus *Heterodea* Nyl. Lichenologist 10: 13–25.

[49] Verdon D, Elix JA (1986) *Myelorrhiza*, a new Australian lichen genus from North Queensland. Brunonia 9: 193–214.

[50] Zhou QM, Wei JC, Ahti T, Stenroos S, Hognabba F (2006) The systematic position of *Gymnoderma* and *Cetradonia* based on SSU rDNA sequences. Journal of the Hattori Botanical Laboratory: 871–880.

[51] Parnmen S, Lumbsch HT (2012) New combinations in the genus *Cladia* . Lichenologist 44: 297–298.

[52] Ahti T (2000) Cladoniaceae. Flora Neotropica 78: 1–362.

[53] Kantvilas G, Elix JA (1987) A new species of *Cladia* (lichenized Ascomycotina) from Tasmania. Mycotaxon 29: 199–205.

[54] Filson R (1992) Cladiaceae. Flora of Australia 54: 101–107.

[55] Galloway DJ (1985) Flora of New Zealand Lichens: P. D. Hasselberg, Government Printer, Wellington. lxxiii + 662 p.

[56] Avise JC, Ball RM (1990) Principles of genealogical concordance in species concepts and biological taxonomy. Oxford Surveys in Evolutionary Biology 7: 45–67.

[57] Dettman JR, Jacobson DJ, Turner E, Pringle A, Taylor JW (2003) Reproductive isolation and phylogenetic divergence in *Neurospora*: Comparing methods of species recognition in a model eukaryote. Evolution 57: 2721–2741. 14761052 10.1111/j.0014-3820.2003.tb01515.x

[58] Dettman JR, Jacobson DJ, Taylor JW (2003) A multilocus genealogical approach to phylogenetic species recognition in the model eukaryote *Neurospora* . Evolution 57: 2703–2720. 14761051 10.1111/j.0014-3820.2003.tb01514.x

[59] Pringle A, Baker DM, Platt JL, Wares JP, Latge JP, et al (2005) Cryptic speciation in the cosmopolitan and clonal human pathogenic fungus *Aspergillus fumigatus* . Evolution 59: 1886–1899. 16261727

[60] Knowles LL, Carstens BC (2007) Delimiting Species without Monophyletic Gene Trees. Systematic Biology 56: 887–895. 18027282 10.1080/10635150701701091

[61] Hickerson MJ, Meyer CP, Moritz C (2006) DNA barcoding will often fail to discover new animal species over broad parameter space. Systematic Biology 55: 729–739. 17060195 10.1080/10635150600969898

[62] Pons J, Barraclough TG, Gomez-Zurita J, Cardoso A, Duran DP, et al (2006) Sequence-based species delimitation for the DNA taxonomy of undescribed insects. Systematic Biology 55: 595–609. 16967577 10.1080/10635150600852011

[63] Monaghan MT, Wild R, Elliot M, Fujisawa T, Balke M, et al (2009) Accelerated Species Inventory on Madagascar Using Coalescent-Based Models of Species Delineation. Systematic Biology 58: 298–311. 20525585 10.1093/sysbio/syp027

[64] Gattolliat JL, Monaghan MT (2010) DNA-based association of adults and larvae in Baetidae (Ephemeroptera) with the description of a new genus *Adnoptilum* in Madagascar. Journal of the North American Benthological Society 29: 1042–1057.

[65] Birky CW, Ricci C, Melone G, Fontaneto D (2011) Integrating DNA and morphological taxonomy to describe diversity in poorly studied microscopic animals: new species of the genus *Abrochtha* Bryce, 1910 (Rotifera: Bdelloidea: Philodinavidae). Zoological Journal of the Linnean Society 161: 723–734.

[66] Lucentini L, Rebora M, Puletti ME, Gigliarelli L, Fontaneto D, et al (2011) Geographical and seasonal evidence of cryptic diversity in the *Baetis rhodani* complex (Ephemeroptera, Baetidae) revealed by means of DNA taxonomy. Hydrobiologia 673: 215–228.

[67] Pons J, Fujisawa T, Claridge EM, Savill RA, Barraclough TG, et al (2011) Deep mtDNA subdivision within Linnean species in an endemic radiation of tiger beetles from New Zealand (genus *Neocicindela*). Molecular Phylogenetics and Evolution 59: 251–262. 21338699 10.1016/j.ympev.2011.02.013

[68] Vuataz L, Sartori M, Wagner A, Monaghan MT (2011) Toward a DNA Taxonomy of Alpine *Rhithrogena* (Ephemeroptera: Heptageniidae) Using a Mixed Yule-Coalescent Analysis of Mitochondrial and Nuclear DNA. Plos One 6. 10.1371/journal.pone.0019728 PMC3096624 21611178

[69] Lorion J, Buge B, Cruaud C, Samadi S (2010) New insights into diversity and evolution of deep-sea Mytilidae (Mollusca: Bivalvia). Molecular Phylogenetics and Evolution 57: 71–83. 20558305 10.1016/j.ympev.2010.05.027

[70] Fontaneto D, Kaya M, Herniou EA, Barraclough TG (2009) Extreme levels of hidden diversity in microscopic animals (Rotifera) revealed by DNA taxonomy. Molecular Phylogenetics and Evolution 53: 182–189. 19398026 10.1016/j.ympev.2009.04.011

[71] Pages M, Chaval Y, Herbreteau V, Waengsothorn S, Cosson JF, et al (2010) Revisiting the taxonomy of the Rattini tribe: a phylogeny-based delimitation of species boundaries. BMC Evolutionary Biology 10: 184. 20565819 10.1186/1471-2148-10-184 PMC2906473

[72] Carstens BC, Dewey TA (2010) Species delimitation using a combined coalescent and information-theoretic approach: an example from North American *Myotis* bats. Systematic Biology 59: 400–414. 20547777 10.1093/sysbio/syq024 PMC2885268

[73] Printzen C, Ekman S, Tønsberg T (2003) Phylogeography of *Cavernularia hultenii*: evidence of slow genetic drift in a widely disjunct lichen. Molecular Ecology 12: 1473–1486. 12755876 10.1046/j.1365-294x.2003.01812.x

[74] Printzen C, Ekman S (2002) Genetic variability and its geographical distribution in the widely disjunct *Cavernularia hultenii* . Lichenologist 34: 101–111.

[75] Fernandez-Mendoza F, Domaschke S, Garcia MA, Jordan P, Martin MP, et al (2011) Population structure of mycobionts and photobionts of the widespread lichen *Cetraria aculeata* . Molecular Ecology 20: 1208–1232. 21324011 10.1111/j.1365-294X.2010.04993.x

[76] Galloway DJ (1977) Additional notes on the lichen genus *Cladia* Nyl., in New Zealand. Nova Hedwigia 28: 475–486.

[77] Schmitt I, Lumbsch HT (2004) Molecular phylogeny of the Pertusariaceae supports secondary chemistry as an important systematic character set in lichen-forming ascomycetes. Molecular Phylogenetics and Evolution 33: 43–55. 15324838 10.1016/j.ympev.2004.04.014

[78] Tehler A, Källersjö M (2001) *Parmeliopsis ambigua* and *P. hyperopta* (Parmeliaceae): species or chemotypes? Lichenologist 33: 403–408.

[79] Blanco O, Crespo A, Divakar PK, Esslinger TL, Hawksworth DL, et al (2004) *Melanelixia* and *Melanohalea*, two new genera segregated from *Melanelia* (Parmeliaceae) based on molecular and morphological data. Mycological Research 108: 873–884. 15449592 10.1017/s0953756204000723

[80] Molina MC, Divakar PK, Millanes AM, Sanchez E, Del-Prado R, et al (2011) *Parmelia sulcata* (Ascomycota: Parmeliaceae), a sympatric monophyletic species complex. Lichenologist 43: 585–601.

[81] Divakar PK, Crespo A, Blanco O, Lumbsch HT (2006) Phylogenetic significance of morphological characters in the tropical *Hypotrachyna* clade of parmelioid lichens (Parmeliaceae, Ascomycota). Molecular Phylogenetics and Evolution 40: 448–458. 16647864 10.1016/j.ympev.2006.03.024

[82] Articus K, Mattsson JE, Tibell L, Grube M, Wedin M (2002) Ribosomal DNA and beta-tubulin data do not support the separation of the lichens *Usnea florida* and *U. subfloridana* as distinct species. Mycological Research 106: 412–418.

[83] Buschbom J, Mueller GM (2006) Testing “species pair” hypotheses: Evolutionary processes in the lichen-forming species complex *Porpidia flavocoerulescens* and *Porpidia melinodes* . Molecular Biology and Evolution 23: 574–586. 16306384 10.1093/molbev/msj063

[84] Nelsen MP, Gargas A (2009) Assessing clonality and chemotype monophyly in *Thamnolia* (Icmadophilaceae). Bryologist 112: 42–53.

[85] Velmala S, Myllys L, Halonen P, Goward T, Ahti T (2009) Molecular data show that *Bryoria fremontii* and *B. tortuosa* (Parmeliaceae) are conspecific. Lichenologist 41: 231–242.

[86] Myllys L, Velmala S, Holien H, Halonen P, Wang L-S, et al (2011) Phylogeny of the genus *Bryoria* . Lichenologist 43: 617–638.

[87] Miralles A, Vasconcelos R, Perera A, Harris DJ, Carranza S (2011) An integrative taxonomic revision of the Cape Verdean skinks (Squamata, Scincidae). Zoologica Scripta 40: 16–44.

[88] Crawford AJ, Lips KR, Bermingham E (2010) Epidemic disease decimates amphibian abundance, species diversity, and evolutionary history in the highlands of central Panama. Proceedings of the National Academy of Sciences of the United States of America 107: 13777–13782. 20643927 10.1073/pnas.0914115107 PMC2922291

[89] Fontaneto D, Iakovenko N, Eyres I, Kaya M, Wyman M, et al (2011) Cryptic diversity in the genus *Adineta* Hudson & Gosse, 1886 (Rotifera: Bdelloidea: Adinetidae): a DNA taxonomy approach. Hydrobiologia 662: 27–33.

[90] Pino-Bodas R, Rosa Burgaz A, Martin MP, Lumbsch HT (2012) Species delimitations in the *Cladonia cariosa* group (Cladoniaceae, Ascomycota). Lichenologist 44: 121–135.

[91] Molina MC, Del-Prado R, Kumar Divakar P, Sanchez-Mata D, Crespo A (2011) Another example of cryptic diversity in lichen-forming fungi: the new species *Parmelia mayi* (Ascomycota: Parmeliaceae). Organisms Diversity & Evolution 11: 331–342.

[92] Pino-Bodas R, Rosa Burgaz A, Martin MP, Lumbsch HT (2011) Phenotypical plasticity and homoplasy complicate species delimitation in the *Cladonia gracilis* group (Cladoniaceae, Ascomycota). Organisms Diversity & Evolution 11: 343–355.

[93] Leavitt SD, Johnson LA, Goward T, St. Clair LL (2011) Species delimitation in taxonomically difficult lichen-forming fungi: an example from morphologically and chemically diverse *Xanthoparmelia* (Parmeliaceae) in North America. Molecular Phylogenetics and Evolution 60: 317–332. 21627994 10.1016/j.ympev.2011.05.012

[94] Wirtz N, Printzen C, Lumbsch HT (2012) Using haplotype networks, estimation of gene flow and phenotypic characters to understand species delimitation in fungi of a predominantly Antarctic *Usnea* group (Ascomycota, Parmeliaceae). Organisms Diversity & Evolution 12: 17–37.

[95] de Queiroz K (2005) Different species problems and their resolution. Bioessays 27: 1263–1269. 16299765 10.1002/bies.20325

[96] de Queiroz K (2007) Species concepts and species delimitation. Systematic Biology 56: 879–886. 18027281 10.1080/10635150701701083

[97] Pecchia S, Mercatelli E, Vannacci G (2004) Intraspecific diversity within *Diapothe helianthi*: evidence from rDNA intergenic spacer (IGS) sequence analysis. Mycopathologia 157: 317–326. 15180160 10.1023/b:myco.0000024185.66158.7e

[98] Carbone I, Kohn LM (1999) A method for designing primer sets for speciation studies in filamentous ascomycetes. Mycologia 91: 553–556.

[99] Gardes M, Bruns TD (1993) ITS primers with enhanced specificity for basidiomycetes – Application to the identification of mycorrhizae and rusts. Molecular Ecology 2: 113–118. 8180733 10.1111/j.1365-294x.1993.tb00005.x

[100] White TJ, Bruns TD, Lee SB, Taylor JW (1990) Amplification and direct sequencing of fungal ribosomal RNA genes for phylogenetics. In: Innis MA, Gelfand DH, Sninsky JJ, White TJ, editors. PCR Protocols. San Diego: Academic Press. 315–322.

[101] Myllys L, Stenroos S, Thell A (2002) New genes for phylogenetic studies of lichenized fungi: glyceraldehyde-3-phosphate dehydrogenase and beta-tubulin genes. Lichenologist 34: 237–246.

[102] Schmitt I, Crespo A, Divakar PK, Fankhauser J, Herman-Sackett E, et al (2009) New primers for single-copy protein-coding genes for fungal systematics. Persoonia – Molecular Phylogeny and Evolution of Fungi 23: 35–40. 10.3767/003158509X470602 PMC2802727 20198159

[103] Hall TA (1999) BioEdit: a user-friendly biological sequence alignment editor and analysis program for Windows 95/98/NT. Nucleic Acids Symposium Series 41: 95–98.

[104] White FJ, James PW (1985) A new guide to microchemical techniques for the identification of lichen substances. British Lichen Society Bulletin 57 (supplement): 1–41.

[105] Lumbsch HT (2002) Analysis of phenolic products in lichens for identification and taxonomy. In: Kranner I, Beckett R, Varma A, editors. Protocols in Lichenology Culturing, biochemistry, ecophysiology and use in biomonitoring. Berlin: Springer. 281–295.

[106] Feige GB, Lumbsch HT, Huneck S, Elix JA (1993) Identification of lichen substance by a standardized high-performance liquid-chromatographic method. Journal of Chromatography 646: 417–427.

[107] Lutzoni F, Kauff F, Cox C, McLaughlin D, Celio G, et al (2004) Assembling the fungal tree of life: progress, classification, and evolution of subcellular traits. American Journal of Botany 91: 1446–1480. 21652303 10.3732/ajb.91.10.1446

[108] Huelsenbeck JP, Ronquist F (2001) MRBAYES: Bayesian inference of phylogenetic trees. Bioinformatics 17: 754–755. 11524383 10.1093/bioinformatics/17.8.754

[109] Nylander JAA, Ronquist F, Huelsenbeck JP, Nieves-Aldrey JL (2004) Bayesian phylogenetic analysis of combined data. Systematic Biology 53: 47–67. 14965900 10.1080/10635150490264699

[110] Nylander JAA, Wilgenbusch JC, Warren DL, Swofford DL (2007) AWTY (Are We There Yet?): a system for graphical exploration of MCMC convergence in Bayesian phylogenetics. Bioinformatics 24: 581–583. 17766271 10.1093/bioinformatics/btm388

[111] Stamatakis A (2006) RAxML-VI-HPC: Maximum Likelihood-based Phylogenetic Analyses with Thousands of Taxa and Mixed Models. Bioinformatics 22: 2688–2690. 16928733 10.1093/bioinformatics/btl446

[112] Felsenstein J (1985) Confidence-limits on phylogenies – an approach using the bootstrap. Evolution 39: 783–791. 28561359 10.1111/j.1558-5646.1985.tb00420.x

[113] Page RDM (1996) Treeview: an application to display phylogenetic trees on personal computers. Computer Applied Biosciences 12: 357–358. 10.1093/bioinformatics/12.4.357 8902363

[114] Paradis E (2006) Analysis of Phylogenetics and Evolution with R; Gentleman R, Hornik K, Parmigiani G, editors. New York: Springer Science. 211 p.

[115] Sanderson MJ (2002) Estimating absolute rates of molecular evolution and divergence times: a penalized likelihood approach. Molecular Biology and Evolution 19: 101–109. 11752195 10.1093/oxfordjournals.molbev.a003974

[116] Thorne JL, Kishino H (2002) Divergence time and evolutionary rate estimation with multilocus data. Systematic Biology 51: 689–702. 12396584 10.1080/10635150290102456

[117] Nee S, Mooers AO, Harvey PH (1992) Tempo and mode of evolution revealed from molecular phylogenies. Proceedings of the National Academy of Sciences of the United States of America 89: 8322–8326. 1518865 10.1073/pnas.89.17.8322 PMC49910

[118] Heled J, Drummond AJ (2010) Bayesian Inference of Species Trees from Multilocus Data. Molecular Biology and Evolution 27: 570–580. 19906793 10.1093/molbev/msp274 PMC2822290

[119] Drummond AJ, Rambaut A (2007) Beast: Bayesian evoluionary analysis by sampling trees. BMC Evolutionary Biology 7: 214. 17996036 10.1186/1471-2148-7-214 PMC2247476

[120] Kubatko LS, Carstens BC, Knowles LL (2009) STEM: species tree estimation using maximum likelihood for gene trees under coalescence. Bioinformatics 25: 971–973. 19211573 10.1093/bioinformatics/btp079

[121] Stamatakis A, Hoover P, Rougemont J (2008) A Rapid Bootstrap Algorithm for the RAxML Web Servers. Systematic Biology 57: 758–771. 18853362 10.1080/10635150802429642

[122] Paradis E, Claude J, Strimmer K (2004) APE: analyses of phylogenetics and evolution in R language. Bioinformatics 20: 289–290. 14734327 10.1093/bioinformatics/btg412

[123] Maddison W, Maddison D (2011) Mesquite: a modular system for evolutionary analysis. Mesquite website. Available: http://mesquiteproject.org. Accessed 2012 Nov 19. Version 2.75 ed.

