# Supplementary material for: Using Phylogenetic and Coalescent Methods to Understand the Species Diversity in the Cladia aggregata Complex (Ascomycota, Lecanorales)
Source: PLoS One. 2012 Dec 18;7(12):e52245. doi: 10.1371/journal.pone.0052245 (PMC3525555; doi:10.1371/journal.pone.0052245)
Supplement: Figure S1 — Majority-consensus tree depicting relationships within the Cladia aggregata complex. A) ITS rDNA, B) IGS rDNA, C) protein-coding GAPDH and D) protein-coding Mcm7 sequences. (DOC) [file pone.0052245.s001.doc]

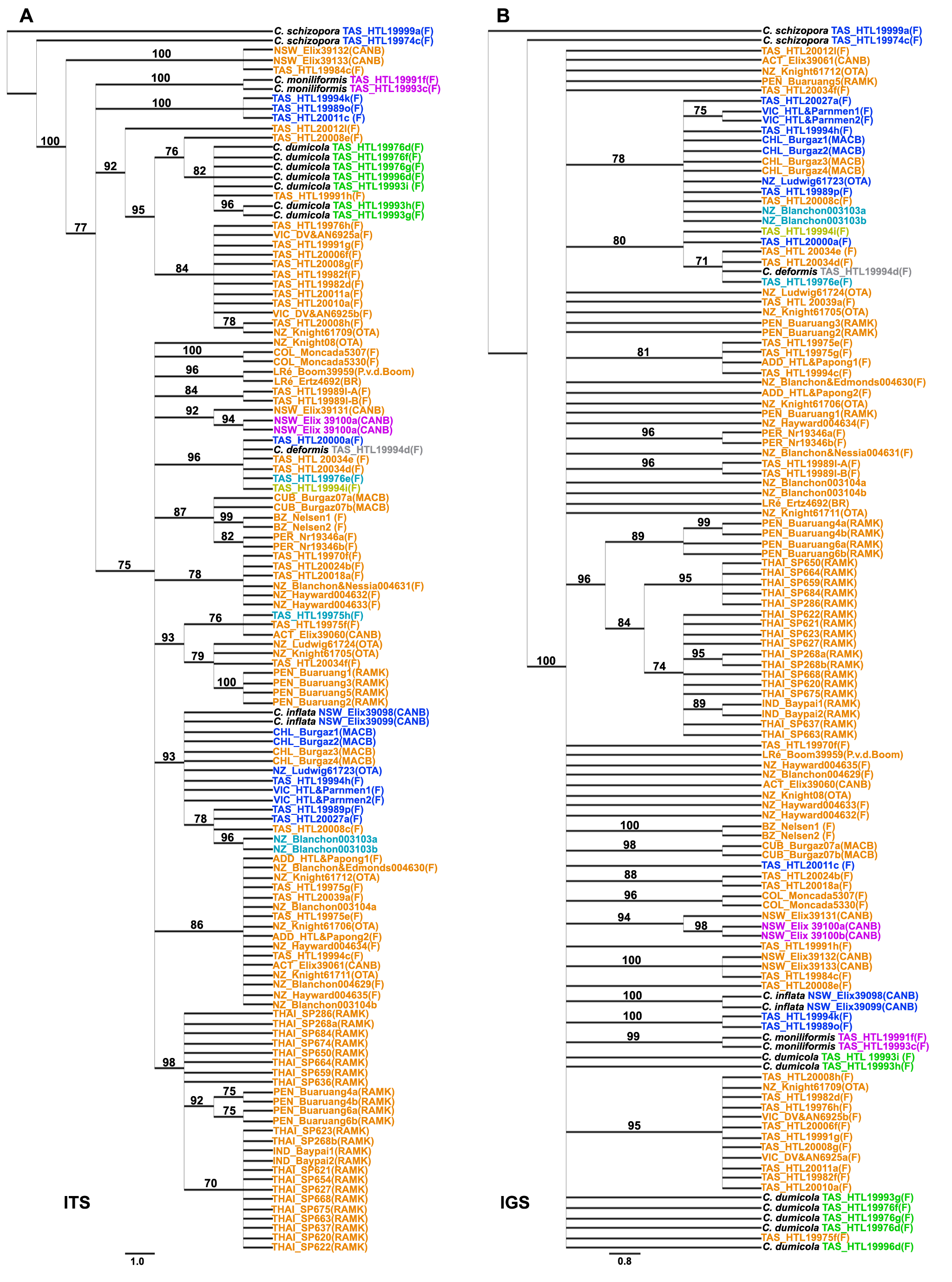


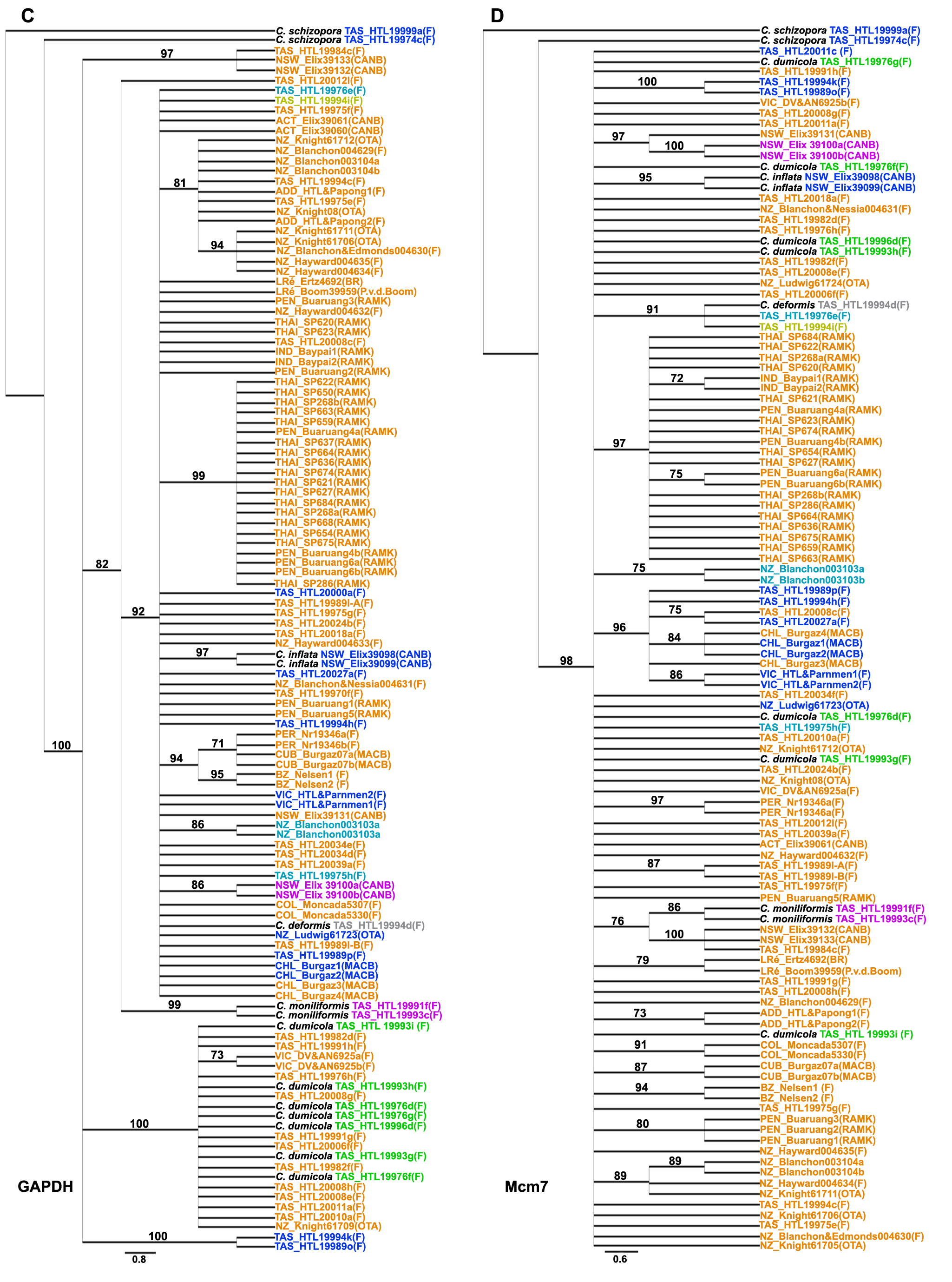


**Supplementary Figure** **S1.**  **Majority-consensus tree depicting relationships within the *Cladia aggregata* complex.** A) ITS rDNA, B) IGS rDNA, C) protein-coding GAPDH and D) protein-coding Mcm7 sequences.
