# Supplementary material for: Using Phylogenetic and Coalescent Methods to Understand the Species Diversity in the Cladia aggregata Complex (Ascomycota, Lecanorales)
Source: PLoS One. 2012 Dec 18;7(12):e52245. doi: 10.1371/journal.pone.0052245 (PMC3525555; doi:10.1371/journal.pone.0052245)
Supplement: Figure S2 — Species trees of the Cladia aggregata species complex inferred from four loci using *Beast. Posterior probabilities are indicated next to each node. All individuals were assigned a priori to either nine species (A) or 12 species (B). (DOC) [file pone.0052245.s002.doc]

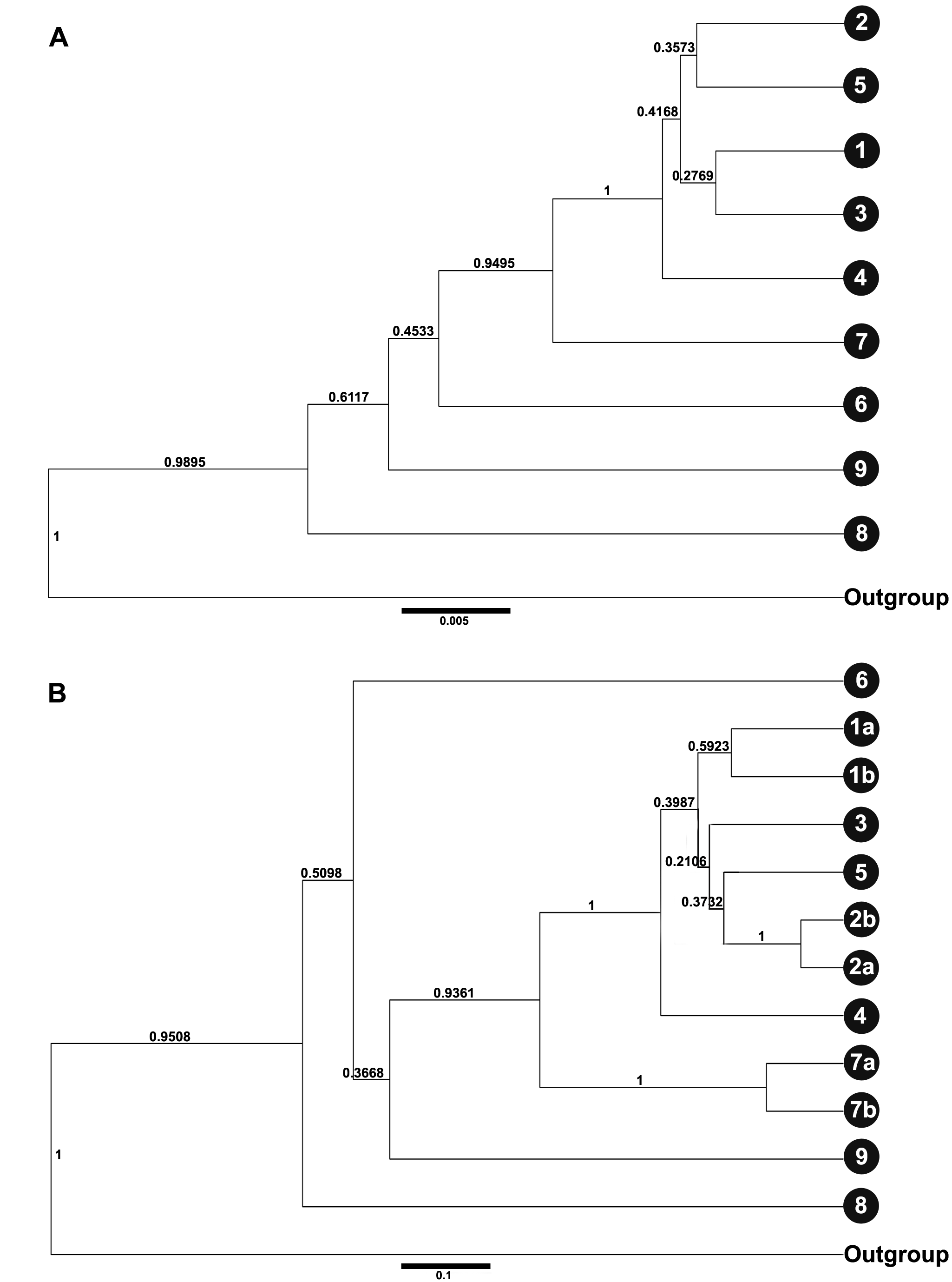


**Supplementary Figure S2.**  **Species trees of the *Cladia aggregata* species complex inferred from four loci using *Beast.** Posterior probabilities are indicated next to each node. All individuals were assigned a priori to either nine species (A) or 12 species (B).
