# Supplementary material for: Using Phylogenetic and Coalescent Methods to Understand the Species Diversity in the Cladia aggregata Complex (Ascomycota, Lecanorales)
Source: PLoS One. 2012 Dec 18;7(12):e52245. doi: 10.1371/journal.pone.0052245 (PMC3525555; doi:10.1371/journal.pone.0052245)
Supplement: Table S1 — Specimens used in this study. New sequences are indicated in bold. (DOC) [file pone.0052245.s003.doc]

| **Supplementary Table S1 Specimens used in this study.** New sequences are indicated in bold. | | | | | |
| --- | --- | --- | --- | --- | --- |
| **Taxa** | **Locality and herbarium accession number** | **Genbank accession number** | | | |
|  |  | **IGS** | **nuITS** | **GAPDH** | **Mcm7** |
| *Cladia aggregata* (Sw.) Nyl. | Australia: New South Wales, Elix39133 (CANB) | **KC148235** | **JN115219** | **JN115102** | **KC148382** |
| *C. aggregata* (Sw.) Nyl. | Australia: Australian Capital Territory, Elix39060 (CANB) | **KC148236** | **JN115218** | **JN115103** | **-** |
| *C. aggregata* (Sw.) Nyl. | Australia: Australian Capital Territory, Elix 39061 (CANB) | **KC148237** | **JN115220** | **JN115104** | **KC148383** |
| *C. aggregata* (Sw.) Nyl. | Australia: New South Wales, Elix 39100a (CANB) | **KC148238** | **JN115223** | **JN115105** | **KC148386** |
| *C. aggregata* (Sw.) Nyl. | Australia: New South Wales, Elix 39100b (CANB) | **KC148239** | **JN115224** | **JN115106** | **KC148387** |
| *C. aggregata* (Sw.) Nyl. | Australia: New South Wales, Elix 39131 (CANB) | **KC148240** | **JN115226** | **JN115100** | **KC148388** |
| *C. aggregata* (Sw.) Nyl. | Australia: New South Wales, Elix 39132 (CANB) | **KC148241** | **JN115227** | **JN115099** | **KC148389** |
| *C. aggregata* (Sw.) Nyl. | Australia: Adelaide, HTL & Papong1 (F) | **KC148332** | **KC148357** | **KC148212** | **KC148396** |
| *C. aggregata* (Sw.) Nyl. | Australia: Adelaide, HTL & Papong2 (F) | **KC148333** | **KC148358** | **KC148213** | **KC148398** |
| *C. aggregata* (Sw.) Nyl. | Australia: Victoria, DV&AN6925a (F) | **KC148355** | **KC148380** | **KC148233** | **KC148415** |
| *C. aggregata* (Sw.) Nyl. | Australia: Victoria, DV&AN6925b (F) | **KC148356** | **KC148381** | **KC148234** | **KC148416** |
| *C. aggregata* (Sw.) Nyl. | Australia: Victoria, HTL&Parnmen1 (F) | **KC148334** | **KC148359** | **KC148214** | **KC148397** |
| *C. aggregata* (Sw.) Nyl. | Australia: Victoria, HTL&Parnmen2 (F) | **KC148335** | **KC148360** | **KC148215** | **KC148399** |
| *C. aggregata* (Sw.) Nyl. | Australia: Tasmania, HTL20000a (F) | **KC148242** | **JN115314** | **JN115188** | **-** |
| *C. aggregata* (Sw.) Nyl. | Australia: Tasmania, HTL20018a (F) | **KC148243** | **JN115316** | **JN115189** | **KC148484** |
| *C. aggregata* (Sw.) Nyl. | Australia: Tasmania, HTL19970f (F) | **KC148244** | GQ500914 | **JN115098** | **-** |
| *C. aggregata* (Sw.) Nyl. | Australia: Tasmania, HTL19975e (F) | **KC148245** | **JN115326** | **JN115190** | **KC148491** |
| *C. aggregata* (Sw.) Nyl. | Australia: Tasmania, HTL19975f (F) | **KC148246** | **JN115270** | **JN115166** | **KC148453** |
| *C. aggregata* (Sw.) Nyl. | Australia: Tasmania, HTL19975g (F) | **KC148247** | **JN115273** | **JN115191** | **KC148455** |
| *C. aggregata* (Sw.) Nyl. | Australia: Tasmania, HTL19975h (F) | **-** | **JN115274** | **JN115113** | **KC148456** |
| *C. aggregata* (Sw.) Nyl. | Australia: Tasmania, HTL19976e (F) | **KC148248** | **JN115282** | **JN115192** | **KC148462** |
| *C. aggregata* (Sw.) Nyl. | Australia: Tasmania, HTL19976h (F) | **KC148249** | **JN115283** | **JN115097** | **KC148463** |
| *C. aggregata* (Sw.) Nyl. | Australia: Tasmania, HTL19982d (F) | **KC148250** | **JN115279** | **JN115107** | **KC148459** |
| *C. aggregata* (Sw.) Nyl. | Australia: Tasmania, HTL19982f (F) | **KC148251** | **JN115280** | **JN115109** | **KC148460** |
| *C. aggregata* (Sw.) Nyl. | Australia: Tasmania, HTL19984c (F) | **KC148252** | **JN115275** | **JN115110** | **KC148457** |
| *C. aggregata* (Sw.) Nyl. | Australia: Tasmania, HTL19989lA (F) | **KC148253** | **JN115312** | **JN115193** | **KC148482** |
| *C. aggregata* (Sw.) Nyl. | Australia: Tasmania, HTL19989lB (F) | **KC148254** | **JN115313** | **JN115194** | **KC148483** |
| *C. aggregata* (Sw.) Nyl. | Australia: Tasmania, HTL19989o (F) | **KC148255** | **JN115303** | **JN115196** | **KC148476** |
| *C. aggregata* (Sw.) Nyl. | Australia: Tasmania, HTL19989p (F) | **KC148256** | **JN115317** | **JN115197** | **KC148485** |
| *C. aggregata* (Sw.) Nyl. | Australia: Tasmania, HTL19991g (F) | **KC148257** | **JN115320** | **JN115198** | **KC148486** |
| *C. aggregata* (Sw.) Nyl. | Australia: Tasmania, HTL19991h (F) | **KC148258** | **JN115327** | **JN115199** | **KC148492** |
| *C. aggregata* (Sw.) Nyl. | Australia: Tasmania, HTL19994c (F) | **KC148259** | GQ500917 | **JN115114** | HM441287 |
| *C. aggregata* (Sw.) Nyl. | Australia: Tasmania, HTL19994h (F) | **KC148260** | **JN115318** | **JN115204** | **KC148452** |
| *C. aggregata* (Sw.) Nyl. | Australia: Tasmania, HTL19994k (F) | **KC148261** | **JN115322** | **JN115205** | **KC148488** |
| *C. aggregata* (Sw.) Nyl. | Australia: Tasmania, HTL19994i (F) | **KC148262** | **JN115294** | **JN115115** | **KC148473** |
| *C. aggregata* (Sw.) Nyl. | Australia: Tasmania, HTL20006f (F) | **KC148263** | **JN115305** | **JN115207** | **KC148478** |
| *C. aggregata* (Sw.) Nyl. | Australia: Tasmania, HTL20008c (F) | **KC148264** | **JN115324** | **JN115208** | **KC148489** |
| *C. aggregata* (Sw.) Nyl. | Australia: Tasmania, HTL20008e (F) | **KC148265** | **JN115285** | **JN115116** | **KC148466** |
| *C. aggregata* (Sw.) Nyl. | Australia: Tasmania, HTL20008g (F) | **KC148266** | **JN115288** | **JN115118** | **KC148468** |
| *C. aggregata* (Sw.) Nyl. | Australia: Tasmania, HTL20008h (F) | **KC148267** | **JN115287** | **JN115119** | **KC148467** |
| *C. aggregata* (Sw.) Nyl. | Australia: Tasmania, HTL20010a (F) | **KC148268** | **JN115284** | **JN115210** | **KC148465** |
| *C. aggregata* (Sw.) Nyl. | Australia: Tasmania, HTL20011a (F) | **KC148269** | **JN115289** | **JN115120** | **KC148469** |
| *C. aggregata* (Sw.) Nyl. | Australia: Tasmania, HTL20011c (F) | **KC148270** | **JN115301** | - | **KC148475** |
| *C. aggregata* (Sw.) Nyl. | Australia: Tasmania, HTL20012l (F) | **KC148271** | **JN115311** | **JN115214** | **KC148481** |
| *C. aggregata* (Sw.) Nyl. | Australia: Tasmania, HTL20024b (F) | **KC148272** | **JN115308** | **JN115215** | **KC148480** |
| *C. aggregata* (Sw.) Nyl. | Australia: Tasmania, HTL20027a (F) | **KC148273** | **JN115307** | **JN115216** | **KC148479** |
| *C. aggregata* (Sw.) Nyl. | Australia: Tasmania, HTL20034d (F) | **KC148274** | **JN115302** | **JN115168** | **-** |
| *C. aggregata* (Sw.) Nyl. | Australia: Tasmania, HTL20034e (F) | **KC148275** | **JN115299** | **JN115167** | **-** |
| *C. aggregata* (Sw.) Nyl. | Australia: Tasmania, HTL20039a (F) | **KC148276** | **JN115292** | **JN115169** | **KC148471** |
| *C. aggregata* (Sw.) Nyl. | Australia: Tasmania, HTL20034f (F) | **KC148277** | **JN115321** | **-** | **KC148487** |
| *C. aggregata* (Sw.) Nyl. | Brazil, BZ1Nelsen (F) | **KC148278** | **JN115228** | **JN115122** | **KC148390** |
| *C. aggregata* (Sw.) Nyl. | Brazil, BZ2Nelsen (F) | **KC148279** | **JN115229** | **JN115123** | **KC148391** |
| *C. aggregata* (Sw.) Nyl. | Peru, Nr19346b1 (F) | **KC148313** | **JN115250** | **JN115153** | **KC148434** |
| *C. aggregata* (Sw.) Nyl. | Peru, Nr19346b2 (F) | **KC148314** | **JN115251** | **JN115154** | **KC148435** |
| *C. aggregata* (Sw.) Nyl. | Cuba, BurgazCUB1 (MACB) | **KC148280** | **JN115234** | **JN115124** | **KC148418** |
| *C. aggregata* (Sw.) Nyl. | Cuba, BurgazCUB2 (MACB) | **KC148281** | **JN115235** | **JN115125** | **KC148419** |
| *C. aggregata* (Sw.) Nyl. | Columbia, Moncada5307 (F) | **KC148351** | **KC148376** | **KC148229** | **KC148408** |
| *C. aggregata* (Sw.) Nyl. | Columbia, Moncada 5330 (F) | **KC148352** | **KC148377** | **KC148230** | **KC148409** |
| *C. aggregata* (Sw.) Nyl. | Chile, BurgazCHL1 (MACB) | **KC148317** | **JN115232** | **JN115180** | **KC148392** |
| *C. aggregata* (Sw.) Nyl. | Chile, BurgazCHL2 (MACB) | **KC148318** | **JN115233** | **JN115181** | **KC148393** |
| *C. aggregata* (Sw.) Nyl. | Chile, BurgazCHL3 (MACB) | **KC148316** | **JN115230** | **JN115178** | **KC148394** |
| *C. aggregata* (Sw.) Nyl. | Chile, BurgazCHL4 (MACB) | **KC148315** | **JN115231** | **JN115179** | **KC155579** |
| *C. aggregata* (Sw.) Nyl. | La Réunion, Ertz4692 (BR) | **KC148353** | **KC148378** | **KC148231** | **KC148407** |
| *C. aggregata* (Sw.) Nyl. | La Réunion, Boom39959 (P.v.d.Boom) | **KC148354** | **KC148379** | **KC148232** | **KC148400** |
| *C. aggregata* (Sw.) Nyl. | India, Baypai1 (RAMK) | **KC148282** | **JN115236** | **JN115126** | **KC148420** |
| *C. aggregata* (Sw.) Nyl. | India, Baypai2 (RAMK) | **KC148283** | **JN115237** | **JN115127** | **KC148421** |
| *C. aggregata* (Sw.) Nyl. | New Zealand, Blanchon003103a (F) | **KC148284** | **JN115240** | **JN115128** | **KC148424** |
| *C. aggregata* (Sw.) Nyl. | New Zealand, Blanchon003103b (F) | **KC148285** | **JN115241** | **JN115172** | **KC148425** |
| *C. aggregata* (Sw.) Nyl. | New Zealand, Blanchon003104a (F) | **KC148286** | **JN115238** | **JN115129** | **KC148422** |
| *C. aggregata* (Sw.) Nyl. | New Zealand, Blanchon003104b (F) | **KC148287** | **JN115239** | **JN115130** | **KC148423** |
| *C. aggregata* (Sw.) Nyl. | New Zealand, Blanchon004629 (F) | **KC148336** | **KC148361** | **KC148216** | **KC148401** |
| *C. aggregata* (Sw.) Nyl. | New Zealand, Blanchon & Edmonds004630 (F) | **KC148337** | **KC148362** | **KC148217** | **KC148402** |
| *C. aggregata* (Sw.) Nyl. | New Zealand, Blanchon & Nessia004631 (F) | **KC148338** | **KC148363** | **KC148218** | **KC148403** |
| *C. aggregata* (Sw.) Nyl. | New Zealand, Hayward004632 (F) | **KC148339** | **KC148364** | **KC148219** | **KC148404** |
| *C. aggregata* (Sw.) Nyl. | New Zealand, Hayward004633 (F) | **KC148340** | **KC148365** | **KC148220** | **-** |
| *C. aggregata* (Sw.) Nyl. | New Zealand, Hayward004634 (F) | **KC148341** | **KC148366** | **KC148221** | **KC148405** |
| *C. aggregata* (Sw.) Nyl. | New Zealand, Hayward004635 (F) | **KC148342** | **KC148367** | **KC148222** | **KC148406** |
| *C. aggregata* (Sw.) Nyl. | New Zealand, Knight61705 (OTA) | **KC148343** | **KC148368** | **-** | **KC148410** |
| *C. aggregata* (Sw.) Nyl. | New Zealand, Knight61706 (OTA) | **KC148344** | **KC148369** | **KC148223** | **KC148411** |
| *C. aggregata* (Sw.) Nyl. | New Zealand, Knight61709 (OTA) | **KC148345** | **KC148370** | **KC148224** | **-** |
| *C. aggregata* (Sw.) Nyl. | New Zealand, Knight61711 (OTA) | **KC148346** | **KC148371** | **KC148225** | **KC148395** |
| *C. aggregata* (Sw.) Nyl. | New Zealand, Knight61712 (OTA) | **KC148347** | **KC148372** | **KC148226** | **KC148412** |
| *C. aggregata* (Sw.) Nyl. | New Zealand, Ludwig61723 (OTA) | **KC148348** | **KC148373** | **KC148227** | **KC148413** |
| *C. aggregata* (Sw.) Nyl. | New Zealand, Ludwig61724 (OTA) | **KC148349** | **KC148374** | **-** | **KC148414** |
| *C. aggregata* (Sw.) Nyl. | New Zealand, Knight08 (OTA) | **KC148350** | **KC148375** | **KC148228** | **KC148417** |
| *C. aggregata* (Sw.) Nyl. | Thailand: Phitsanulok Province, SP268 (RAMK) | **KC148288** | EU091338 | **JN115131** | **KC148436** |
| *C. aggregata* (Sw.) Nyl. | Thailand: Phitsanulok Province, SP268b (RAMK) | **KC148289** | **JN115252** | **JN115170** | **KC148437** |
| *C. aggregata* (Sw.) Nyl. | Thailand: Loei Province, SP620 (RAMK) | **KC148290** | **JN115253** | **JN115132** | **KC148439** |
| *C. aggregata* (Sw.) Nyl. | Thailand: Loei Province, SP622 (RAMK) | **KC148291** | **JN115255** | **JN115133** | **KC148441** |
| *C. aggregata* (Sw.) Nyl. | Thailand: Loei Province, SP623 (RAMK) | **KC148292** | **JN115256** | **JN115134** | **KC148442** |
| *C. aggregata* (Sw.) Nyl. | Thailand: Loei Province, SP286 (RAMK) | **KC148293** | EU113276 | **JN115135** | **KC148438** |
| *C. aggregata* (Sw.) Nyl. | Thailand: Loei Province, SP627 (RAMK) | **KC148294** | **JN115257** | **JN115136** | **KC148443** |
| *C. aggregata* (Sw.) Nyl. | Thailand: Loei Province, SP636 (RAMK) | **-** | **JN115258** | **JN115137** | **KC148444** |
| *C. aggregata* (Sw.) Nyl. | Thailand: Loei Province, SP637 (RAMK) | **KC148295** | **JN115259** | **JN115138** | **-** |
| *C. aggregata* (Sw.) Nyl. | Thailand: Loei Province, SP650 (RAMK) | **KC148296** | **JN115260** | **JN115173** | **-** |
| *C. aggregata* (Sw.) Nyl. | Thailand: Loei Province, SP654 (RAMK) | **-** | **JN115261** | **JN115139** | **KC148445** |
| *C. aggregata* (Sw.) Nyl. | Thailand: Loei Province, SP659 (RAMK) | **KC148297** | **JN115262** | **JN115140** | **KC148446** |
| *C. aggregata* (Sw.) Nyl. | Thailand: Loei Province, SP663 (RAMK) | **KC148298** | **JN115263** | **JN115165** | **KC148447** |
| *C. aggregata* (Sw.) Nyl. | Thailand: Loei Province, SP664 (RAMK) | **KC148299** | **JN115264** | **JN115141** | **KC148448** |
| *C. aggregata* (Sw.) Nyl. | Thailand: Loei Province, SP668 (RAMK) | **KC148300** | **JN115265** | **JN115174** | **-** |
| *C. aggregata* (Sw.) Nyl. | Thailand: Loei Province, SP675 (RAMK) | **KC148301** | **JN115268** | **JN115142** | **KC148450** |
| *C. aggregata* (Sw.) Nyl. | Thailand: Loei Province, SP684 (RAMK) | **KC148302** | **JN115269** | **JN115143** | **KC148451** |
| *C. aggregata* (Sw.) Nyl. | Thailand: Loei Province, SP621 (RAMK) | **KC148331** | **JN115254** | **JN115184** | **KC148440** |
| *C. aggregata* (Sw.) Nyl. | Thailand: Loei Province, SP674 (RAMK) | **-** | **JN115267** | **JN115185** | **KC148449** |
| *C. aggregata* (Sw.) Nyl. | Malaysia: Penang, PEN1 (RAMK) | **KC148319** | **JN115242** | **JN115155** | **KC148426** |
| *C. aggregata* (Sw.) Nyl. | Malaysia: Penang, PEN2 (RAMK) | **KC148320** | **JN115243** | **JN115156** | **KC148427** |
| *C. aggregata* (Sw.) Nyl. | Malaysia: Penang, PEN3 (RAMK) | **KC148321** | **JN115244** | **JN115157** | **KC148428** |
| *C. aggregata* (Sw.) Nyl. | Malaysia: Penang, PEN4a (RAMK) | **KC148322** | **JN115245** | **JN115158** | **KC148429** |
| *C. aggregata* (Sw.) Nyl. | Malaysia: Penang, PEN4b (RAMK) | **KC148323** | **JN115246** | **JN115182** | **KC148430** |
| *C. aggregata* (Sw.) Nyl. | Malaysia: Penang, PEN5 (RAMK) | **KC148324** | **JN115247** | **JN115159** | **KC148431** |
| *C. aggregata* (Sw.) Nyl. | Malaysia: Penang, PEN6a (RAMK) | **KC148325** | **JN115248** | **JN115160** | **KC148432** |
| *C. aggregata* (Sw.) Nyl. | Malaysia: Penang, PEN6b (RAMK) | **KC148326** | **JN115249** | **JN115183** | **KC148433** |
| *C. deformis* Kantvilas & Elix | Australia: Tasmania, HTL19994d (F) | **KC148303** | GQ500923 | **JN115144** | HM441279 |
| *C. dumicola* Kantvilas & Elix | Australia: Tasmania, HTL19976d (F) | **KC148304** | **JN115277** | **JN115145** | **KC148458** |
| *C. dumicola* Kantvilas & Elix | Australia: Tasmania, HTL19976g (F) | **KC148305** | **JN115272** | **JN115146** | **KC148454** |
| *C. dumicola* Kantvilas & Elix | Australia: Tasmania, HTL19993g (F) | **KC148306** | GQ500915 | **JN115147** | HM441281 |
| *C. dumicola* Kantvilas & Elix | Australia: Tasmania, HTL19993h (F) | **KC148307** | **JN115295** | **JN115148** | **KC148474** |
| *C. dumicola* Kantvilas & Elix | Australia: Tasmania, HTL19993i (F) | **KC148308** | **JN115325** | **JN115176** | **KC148490** |
| *C. dumicola* Kantvilas & Elix | Australia: Tasmania, HTL19996d (F) | **KC148309** | **JN115304** | **JN115177** | **KC148477** |
| *C. dumicola* Kantvilas & Elix | Australia: Tasmania, HTL19976f (F) | **KC148312** | **JN115281** | **JN115152** | **KC148461** |
| *C. inflata* (F.Wilson) D.J. Galloway | Australia: New South Wales, Elix 39098 (CANB) | **KC148310** | **JN115221** | **JN115150** | **KC148384** |
| *C. inflata* (F.Wilson) D.J. Galloway | Australia: New South Wales, Elix 39099 (CANB) | **KC148311** | **JN115222** | **JN115151** | **KC148385** |
| *C. moniliformis* Kantvilas & Elix | Australia: Tasmania, HTL19991f (F) | **KC148327** | GQ500910 | **JN115164** | HM441286 |
| *C. moniliformis* Kantvilas & Elix | Australia: Tasmania, HTL19993c (F) | **KC148328** | **JN115293** | **JN115161** | **KC148472** |
| *C. schizopora* (Nyl.) Nyl. | Australia: Tasmania, HTL19974c (F) | **KC148329** | GQ500919 | **JN115162** | **KC148464** |
| *C. schizopora* (Nyl.) Nyl. | Australia: Tasmania, HTL19999a (F) | **KC148330** | **JN115291** | **JN115163** | **KC148470** |
