# Supplementary material for: Using Phylogenetic and Coalescent Methods to Understand the Species Diversity in the Cladia aggregata Complex (Ascomycota, Lecanorales)
Source: PLoS One. 2012 Dec 18;7(12):e52245. doi: 10.1371/journal.pone.0052245 (PMC3525555; doi:10.1371/journal.pone.0052245)
Supplement: Table S2 — A data matrix containing morphological and chemical characters of the genus Cladia under study. (DOC) [file pone.0052245.s004.doc]

| **Supplementary Table S2** A data matrix containing morphological and chemical characters of the genus *Cladia* under study | | | | | |
| --- | --- | --- | --- | --- | --- |
| **No.** | **Species** | **Herbarium number** | **1-10** | **11-20** | **21-27** |
| 1 | *Cladia aggregata* (Sw.) Nyl. | Elix 39133 (CANB) | 00000000?? | ???????101 | 0000000 |
| 2 | *C. aggregata* (Sw.) Nyl. | Elix 39060 (CANB) | 00001000?? | ??????0101 | 0000000 |
| 3 | *C. aggregata* (Sw.) Nyl. | Elix 39061(CANB) | 10000101?? | ??????0101 | 0000000 |
| 4 | *C. aggregata* (Sw.) Nyl. | Elix 39100a(CANB) | 00002000?? | ??????0000 | 0000110 |
| 5 | *C. aggregata* (Sw.) Nyl. | Elix 39100b(CANB) | 00002000?? | ??????0000 | 0000110 |
| 6 | *C. aggregata* (Sw.) Nyl. | Elix 39131(CANB) | 0000000001 | 0000110101 | 0000000 |
| 7 | *C. aggregata* (Sw.) Nyl. | Elix 39132(CANB) | 0000000001 | 0000110101 | 0000000 |
| 8 | *C. aggregata* (Sw.) Nyl. | HTL 20000a (F) | 1000010120 | 0001110000 | 1100000 |
| 9 | *C. aggregata* (Sw.) Nyl. | HTL 20018a (F) | 00003020?? | ???????101 | 0000000 |
| 10 | *C. aggregata* (Sw.) Nyl. | HTL 19970f (F) | 00001020?? | ???????101 | 0000000 |
| 11 | *C. aggregata* (Sw.) Nyl. | HTL 19975e (F) | 1000100020 | 0001000101 | 0000000 |
| 12 | *C. aggregata* (Sw.) Nyl. | HTL 19975f (F) | 00101001?? | ??????0101 | 0000000 |
| 13 | *C. aggregata* (Sw.) Nyl. | HTL 19975g (F) | 10001000?? | ???????101 | 0000000 |
| 14 | *C. aggregata* (Sw.) Nyl. | HTL 19975h (F) | 0110100011 | 0101100000 | 1011000 |
| 15 | *C. aggregata* (Sw.) Nyl. | HTL 19976e (F) | 00102000?? | ???????000 | 1011000 |
| 16 | *C. aggregata* (Sw.) Nyl. | HTL 19976h (F) | 00103020?? | ???????101 | 0000000 |
| 17 | *C. aggregata* (Sw.) Nyl. | HTL 19982d (F) | 00000000?? | ???????101 | 0000000 |
| 18 | *C. aggregata* (Sw.) Nyl. | HTL 19982f (F) | 00000020?? | ???????101 | 0000000 |
| 19 | *C. aggregata* (Sw.) Nyl. | HTL 19984c (F) | 01000020?? | ???????101 | 0000000 |
| 20 | *C. aggregata* (Sw.) Nyl. | HTL 19989l-A (F) | 00003020?? | ???????101 | 0000000 |
| 21 | *C. aggregata* (Sw.) Nyl. | HTL 19989l-B (F) | 00003020?? | ???????101 | 0000000 |
| 22 | *C. aggregata* (Sw.) Nyl. | HTL 19989o (F) | 10103030?? | ???????000 | 1100000 |
| 23 | *C. aggregata* (Sw.) Nyl. | HTL 19989p (F) | 10001020?? | ??????0000 | 1100000 |
| 24 | *C. aggregata* (Sw.) Nyl. | HTL 19991g (F) | 10001101?? | ???????101 | 0000000 |
| 25 | *C. aggregata* (Sw.) Nyl. | HTL 19991h (F) | 10100010?? | ???????101 | 0000000 |
| 26 | *C. aggregata* (Sw.) Nyl. | HTL 19994c (F) | 0010102011 | 0101110101 | 0000000 |
| 27 | *C. aggregata* (Sw.) Nyl. | HTL 19994h (F) | 10010111?? | ??????0000 | 1100000 |
| 28 | *C. aggregata* (Sw.) Nyl. | HTL 19994k (F) | 10103030?? | ???????000 | 1100000 |
| 29 | *C. aggregata* (Sw.) Nyl. | HTL 19994i (F) | 10101110?? | ??????0110 | 1011000 |
| 30 | *C. aggregata* (Sw.) Nyl. | HTL 20006f (F) | 0000000020 | 0001110101 | 0000000 |
| 31 | *C. aggregata* (Sw.) Nyl. | HTL 20008c (F) | 00101021?? | ????? ??101 | 0000000 |
| 32 | *C. aggregata* (Sw.) Nyl. | HTL 20008e (F) | 0000000011 | 0000110101 | 0000000 |
| 33 | *C. aggregata* (Sw.) Nyl. | HTL 20008g (F) | 0000002020 | 0001110101 | 0000000 |
| 34 | *C. aggregata* (Sw.) Nyl. | HTL 20008h (F) | 0000000020 | 0000110101 | 0000000 |
| 35 | *C. aggregata* (Sw.) Nyl. | HTL 20010a (F) | 00101021?? | ???????101 | 0000000 |
| 36 | *C. aggregata* (Sw.) Nyl. | HTL 20011a (F) | 10001020?? | ???????101 | 0000000 |
| 37 | *C. aggregata* (Sw.) Nyl. | HTL 20011c (F) | ????? ????0 | 000300?000 | 1100000 |
| 38 | *C. aggregata* (Sw.) Nyl. | HTL 20012l (F) | 1001101010 | 000311?101 | 0000000 |
| 39 | *C. aggregata* (Sw.) Nyl. | HTL 20024b (F) | 00003020?? | ???????101 | 0000000 |
| 40 | *C. aggregata* (Sw.) Nyl. | HTL 20027a (F) | 10001020?? | ???????000 | 1100000 |
| 41 | *C. aggregata* (Sw.) Nyl. | HTL 20034d (F) | 0000100020 | 0101110101 | 0000000 |
| 42 | *C. aggregata* (Sw.) Nyl. | HTL 20034e (F) | 0000100020 | 0001110101 | 0000000 |
| 43 | *C. aggregata* (Sw.) Nyl. | HTL 20039a (F) | 10001010?? | ????? ?0101 | 0000000 |
| 44 | *C. aggregata* (Sw.) Nyl. | HTL 20034f (F) | 10001101?? | ???????101 | 0000000 |
| 45 | *C. aggregata* (Sw.) Nyl. | BZ_Nelsen1 (F) | 00000020?? | ???????101 | 0000000 |
| 46 | *C. aggregata* (Sw.) Nyl. | BZ_Nelsen2 (F) | 00000020?? | ???????101 | 0000000 |
| 47 | *C. aggregata* (Sw.) Nyl. | CUB_Burgaz1 (MACB) | 1000000011 | 0000110101 | 0000000 |
| 48 | *C. aggregata* (Sw.) Nyl. | CUB_Burgaz2 (MACB) | 1000000011 | 0000110101 | 0000000 |
| 49 | *C. aggregata* (Sw.) Nyl. | IN-Baypai 1 | 10000101?? | ???????101 | 0000000 |
| 50 | *C. aggregata* (Sw.) Nyl. | IN-Baypai 2 | 10000101?? | ???????101 | 0000000 |
| 51 | *C. aggregata* (Sw.) Nyl. | Blanchon 003103a | 10000121?? | ???????000 | 1011000 |
| 52 | *C. aggregata* (Sw.) Nyl. | Blanchon 003103b | 10000121?? | ???????000 | 1011000 |
| 53 | *C. aggregata* (Sw.) Nyl. | Blanchon 003104a | 10000000?? | ??????0101 | 0000000 |
| 54 | *C. aggregata* (Sw.) Nyl. | Blanchon 003104b | 10000000?? | ??????0101 | 0000000 |
| 55 | *C. aggregata* (Sw.) Nyl. | SP268-1(RAMK) | 10000101?? | ??????0101 | 0000000 |
| 56 | *C. aggregata* (Sw.) Nyl. | SP268-2(RAMK) | 10000101?? | ??????0101 | 0000000 |
| 57 | *C. aggregata* (Sw.) Nyl. | SP620 (RAMK) | 10001101?? | ??????0101 | 0000000 |
| 58 | *C. aggregata* (Sw.) Nyl. | SP622(RAMK) | 10001101?? | ??????0101 | 0000000 |
| 59 | *C. aggregata* (Sw.) Nyl. | SP623(RAMK) | 10001101?? | ??????0101 | 0000000 |
| 60 | *C. aggregata* (Sw.) Nyl. | SP286(RAMK) | 1000010110 | 0000110101 | 0000000 |
| 61 | *C. aggregata* (Sw.) Nyl. | SP627(RAMK) | 10000101?? | ??????0101 | 0000000 |
| 62 | *C. aggregata* (Sw.) Nyl. | SP636(RAMK) | 10000101?? | ??????0101 | 0000000 |
| 63 | *C. aggregata* (Sw.) Nyl. | SP637(RAMK) | 10000101?? | ??????0101 | 0000000 |
| 64 | *C. aggregata* (Sw.) Nyl. | SP650(RAMK) | 10001101?? | ??????0101 | 0000000 |
| 65 | *C. aggregata* (Sw.) Nyl. | SP654(RAMK) | 10001101?? | ??????0101 | 0000000 |
| 66 | *C. aggregata* (Sw.) Nyl. | SP659(RAMK) | 10001101?? | ??????0101 | 0000000 |
| 67 | *C. aggregata* (Sw.) Nyl. | SP663(RAMK) | 10001101?? | ??????0101 | 0000000 |
| 68 | *C. aggregata* (Sw.) Nyl. | SP664(RAMK) | 10003101?? | ??????0101 | 0000000 |
| 69 | *C. aggregata* (Sw.) Nyl. | SP668(RAMK) | 10003101?? | ??????0101 | 0000000 |
| 70 | *C. aggregata* (Sw.) Nyl. | SP675(RAMK) | 10003101?? | ??????0101 | 0000000 |
| 71 | *C. aggregata* (Sw.) Nyl. | SP684(RAMK) | 10003101?? | ??????0101 | 0000000 |
| 72 | *C. aggregata* (Sw.) Nyl. | SP621(RAMK) | 10001101?? | ??????0101 | 0000000 |
| 73 | *C. aggregata* (Sw.) Nyl. | SP674(RAMK) | 10003101?? | ??????0101 | 0000000 |
| 74 | *C. aggregata* (Sw.) Nyl. | PER_Nr.19346b-1 | 10101000?? | ???????101 | 0000000 |
| 75 | *C. aggregata* (Sw.) Nyl. | PER_Nr.19346b-2 | 10101000?? | ???????101 | 0000000 |
| 76 | *C. aggregata* (Sw.) Nyl. | CHL_Burgaz4 | 10000101?? | ??????0101 | 0000000 |
| 77 | *C. aggregata* (Sw.) Nyl. | CHL_Burgaz3 | 10000101?? | ??????0101 | 0000000 |
| 78 | *C. aggregata* (Sw.) Nyl. | CHL_Burgaz1 | 10101000?? | ???????000 | 1100000 |
| 79 | *C. aggregata* (Sw.) Nyl. | CHL_Burgaz2 | 10101000?? | ??????0000 | 1100000 |
| 80 | *C. aggregata* (Sw.) Nyl. | PEN_Buaruang1(RAMK) | 10000020?? | ???????101 | 0000000 |
| 81 | *C. aggregata* (Sw.) Nyl. | PEN_Buaruang 2(RAMK) | 10001101?? | ???????101 | 0000000 |
| 82 | *C. aggregata* (Sw.) Nyl. | PEN_Buaruang 3(RAMK) | 10001020?? | ???????101 | 0000000 |
| 83 | *C. aggregata* (Sw.) Nyl. | PEN_Buaruang 4a(RAMK) | 10001101?? | ??????0101 | 0000000 |
| 84 | *C. aggregata* (Sw.) Nyl. | PEN_Buaruang 4b(RAMK) | 10001101?? | ??????0101 | 0000000 |
| 85 | *C. aggregata* (Sw.) Nyl. | PEN_Buaruang 5(RAMK) | 10000101?? | ???????101 | 0000000 |
| 86 | *C. aggregata* (Sw.) Nyl. | PEN_Buaruang 6a(RAMK) | 10001020?? | ??????0101 | 0000000 |
| 87 | *C. aggregata* (Sw.) Nyl. | PEN_Buaruang 6b(RAMK) | 10001020?? | ??????0101 | 0000000 |
| 88 | *C. aggregata* (Sw.) Nyl. | HTL&Papong1(F) | 1000101010 | 0000110101 | 0000000 |
| 89 | *C. aggregata* (Sw.) Nyl. | HTL&Papong2(F) | 10100020?? | ??????0101 | 0000000 |
| 90 | *C. aggregata* (Sw.) Nyl. | HTL&Parnmen1(F) | 10000121?? | ??????0000 | 1100000 |
| 91 | *C. aggregata* (Sw.) Nyl. | HTL&Parnmen2(F) | 10000121?? | ??????0000 | 1100000 |
| 92 | *C. aggregata* (Sw.) Nyl. | Blanchon004629(F) | 10002010?? | ??????0101 | 0000000 |
| 93 | *C. aggregata* (Sw.) Nyl. | Blanchon&Edmonds004630(F) | 10100010?? | ???????101 | 0000000 |
| 94 | *C. aggregata* (Sw.) Nyl. | Blanchon&Nessia004631(F) | 00003020?? | ???????101 | 0000000 |
| 95 | *C. aggregata* (Sw.) Nyl. | Hayward004632(F) | 00002000?? | ???????101 | 0000000 |
| 96 | *C. aggregata* (Sw.) Nyl. | Hayward004633(F) | 00000100?? | ???????101 | 0000000 |
| 97 | *C. aggregata* (Sw.) Nyl. | Hayward004634(F) | 0000010100 | 000011?101 | 0000000 |
| 98 | *C. aggregata* (Sw.) Nyl. | Hayward004635(F) | 10000111?? | ???????101 | 0000000 |
| 99 | *C. aggregata* (Sw.) Nyl. | Knight61705(OTA) | 1001111121 | 0011110101 | 0000000 |
| 100 | *C. aggregata* (Sw.) Nyl. | Knight61706(OTA) | 0010002011 | 000111?101 | 0000000 |
| 101 | *C. aggregata* (Sw.) Nyl. | Knight61709(OTA) | 00100101?? | ???????101 | 0000000 |
| 102 | *C. aggregata* (Sw.) Nyl. | Knight61711(OTA) | 0010000001 | 000111?101 | 0000000 |
| 103 | *C. aggregata* (Sw.) Nyl. | Knight61712(OTA) | 1000111111 | 000111?101 | 0000000 |
| 104 | *C. aggregata* (Sw.) Nyl. | Ludwig61723(OTA) | 10001111?? | ???????000 | 1100000 |
| 105 | *C. aggregata* (Sw.) Nyl. | Ludwig61724(OTA) | 1001213101 | 001211?101 | 0000000 |
| 106 | *C. aggregata* (Sw.) Nyl. | Knight08(OTA) | 00000100?? | ???????101 | 0000000 |
| 107 | *C. aggregata* (Sw.) Nyl. | Moncada 5307(F) | 1001211110 | 001211?101 | 0000000 |
| 108 | *C. aggregata* (Sw.) Nyl. | Moncada 5330(F) | 1001211110 | 001211?101 | 0000000 |
| 109 | *C. aggregata* (Sw.) Nyl. | Ertz4692(BR) | 10001111?? | ???????101 | 0000000 |
| 110 | *C. aggregata* (Sw.) Nyl. | Boom39959(P.v.d.Boom) | 00012000?? | ???????101 | 0000000 |
| 111 | *C. aggregata* (Sw.) Nyl. | DV&AN6925a(F) | 00000000?? | ???????101 | 0000000 |
| 112 | *C. aggregata* (Sw.) Nyl. | DV&AN6925b(F) | 00000000?? | ???????101 | 0000000 |
| 113 | *C. deformis* Kantvilas & Elix | HTL 19994d (F) | 1000211120 | 0102110000 | 1111000 |
| 114 | *C. dumicola* Kantvilas & Elix | HTL 19976d (F) | 10001121?? | ??????0000 | 0000001 |
| 115 | *C. dumicola* Kantvilas & Elix | HTL 19976g (F) | ????????20 | 000311?000 | 0000001 |
| 116 | *C. dumicola* Kantvilas & Elix | HTL 19993g (F) | 10001121?? | ??????0000 | 0000001 |
| 117 | *C. dumicola* Kantvilas & Elix | HTL 19993h (F) | 00001121?? | ??????0000 | 0000001 |
| 118 | *C. dumicola* Kantvilas & Elix | HTL 19993i (F) | 00003010?? | ???????000 | 0000001 |
| 119 | *C. dumicola* Kantvilas & Elix | HTL 19996d (F) | 00000121?? | ???????000 | 0000001 |
| 120 | *C. dumicola* Kantvilas & Elix | HTL 19976f (F) | 10111010?? | ???????000 | 0000001 |
| 121 | *C. inflata* (F.Wilson) D.J. Galloway | Elix 39098(CANB) | 10001030?? | ??????0000 | 1100000 |
| 122 | *C. inflata* (F.Wilson) D.J. Galloway | Elix 39099 (CANB) | 10001030?? | ??????0000 | 1100000 |
| 123 | *C. moniliformis* Kantvilas & Elix | HTL F19991 (F) | 2111301022 | 1013001000 | 0000110 |
| 124 | *C. moniliformis* Kantvilas & Elix | HTL 19993c (F) | 2111301022 | 1013001000 | 0000110 |
| 125 | *C. schizopora* (Nyl.) Nyl. | HTL 19974c (F) | 1?10202011 | 0002100000 | 1100000 |
| 126 | *C. schizopora* (Nyl.) Nyl. | HTL 19999a (F) | 1?10202011 | 0002100000 | 1100000 |
